# Microbiome—Microbial Metabolome—Cancer Cell Interactions in Breast Cancer—Familiar, but Unexplored

**DOI:** 10.3390/cells8040293

**Published:** 2019-03-29

**Authors:** Edit Mikó, Tünde Kovács, Éva Sebő, Judit Tóth, Tamás Csonka, Gyula Ujlaki, Adrienn Sipos, Judit Szabó, Gábor Méhes, Péter Bai

**Affiliations:** 1 Department of Medical Chemistry, University of Debrecen, 4032 Debrecen, Hungary; miko.edit@med.unideb.hu (E.M.); tunde.kovacs33@gmail.com (T.K.); ujlakigyula15@gmail.com (G.U.); siposadri@med.unideb.hu (A.S.); 2 Department of Microbiology, Faculty of Medicine, University of Debrecen, 4032 Debrecen, Hungary; szabjud@med.unideb.hu; 3 Kenézy Breast Center, Kenézy Gyula County Hospital, 4032 Debrecen, Hungary; seboeva@gmail.com (É.S.); tothjuditdr11@t-online.hu (J.T.); 4 Department of Pathology, Faculty of Medicine, University of Debrecen, 4032 Debrecen, Hungary; csonkatamas84@gmail.com (T.C.); gabor.mehes@med.unideb.hu (G.M.); 5 MTA-DE Lendület Laboratory of Cellular Metabolism, 4032 Debrecen, Hungary; 6 Research Center for Molecular Medicine, Faculty of Medicine, University of Debrecen, 4032 Debrecen, Hungary

**Keywords:** breast cancer, microbiome, estrogen deconjugation, lithocholic acid, secondary bile acids, cadaverine, TGR5, FFAR, TAAR, mitochondrial metabolism, OXPHOS

## Abstract

Breast cancer is a leading cause of death among women worldwide. Dysbiosis, an aberrant composition of the microbiome, characterizes breast cancer. In this review we discuss the changes to the metabolism of breast cancer cells, as well as the composition of the breast and gut microbiome in breast cancer. The role of the breast microbiome in breast cancer is unresolved, nevertheless it seems that the gut microbiome does have a role in the pathology of the disease. The gut microbiome secretes bioactive metabolites (reactivated estrogens, short chain fatty acids, amino acid metabolites, or secondary bile acids) that modulate breast cancer. We highlight the bacterial species or taxonomical units that generate these metabolites, we show their mode of action, and discuss how the metabolites affect mitochondrial metabolism and other molecular events in breast cancer. These metabolites resemble human hormones, as they are produced in a “gland” (in this case, the microbiome) and they are subsequently transferred to distant sites of action through the circulation. These metabolites appear to be important constituents of the tumor microenvironment. Finally, we discuss how bacterial dysbiosis interferes with breast cancer treatment through interfering with chemotherapeutic drug metabolism and availability.

## 1. Breast Cancer, a Leading Cause of Death among Women

Breast cancer is the most common cancer among women, with the estimated age-adjusted annual incidence of breast cancer in Europe in 2012 being 94.2/100,000 [1], with similar numbers in the United States (US) or the United Kingdom (UK) [2,3]. The estimated age-adjusted annual mortality is 23.1/100,000 for breast cancer in Europe [1]. In 2019, in the US, the number of newly diagnosed invasive breast cancer cases is estimated to be 268,000, while the newly diagnosed in situ cases are estimated to be around 62,930 [2]. In 2019, 41,760 women out of these numbers are expected to die of breast cancer in the US [2]. The five year survival of breast cancer is over 80% in developed countries due to screening programs and the consequent early detection [4].

Several risk factors for breast cancer had been described, nevertheless the majority of the newly diagnosed women have no obvious risk factors [3]. The risk for breast cancer increases over the age. The majority of the patients are diagnosed after menopause, after the age of 50. The incidence rates for in situ breast cancer in the UK were the highest in people that were aged 65–69 (2013–2015) [3]. Extended female hormone exposure by the use of hormone-replacement therapy or early menarche or late menopause also increases the risk for breast cancer [3]. Mutations in the *BRCA1* and *BRCA2* genes represent a predisposing factor for breast cancer [5], similarly to a family history of breast cancer or personal history of neoplastic diseases or breast cancer [3] Finally, dense breast is an independent risk factor of breast cancer [1,6]. Physical activity, successful pregnancies, and lactation are protective factors [2,3]. 

In Western countries there are organized screening programs from the age of 40–45 to 65 years of age for women with bi-annual intervals [7,8,9,10]. The first step in screening is mammography, followed by ultrasonography in breast cancer-suspect individuals [1]. The final diagnosis is based on needle biopsy. Breast cancer screening does not reach the whole target population, for example, in Hungary only around 50% of the target population undergoes screening [7].

The treatment schemes for breast cancer include the surgical procedures, chemotherapy, targeted therapy, endocrine-, and radiotherapy. Chemotherapy regimens contain anthracyclines, cyclophosphamides, taxanes, antimetabolites (5-fluorouracil, gemcitabine, capecitabine), and navelbine that targets mitotic tubules [1]. Targeted therapy in breast cancer is used in the management of HER2 positive cases and it involves monoclonal antibodies against the HER2 receptor (trastuzumab, pertuzumab, and trastuzumab-emtansine, in which the humanized HER2 antibody is conjugated to DM1, a tubulin toxin) and the tyrosine kinase inhibitor lapatinib [11]. Endocrine therapy, which involves selective estrogen receptor modulators (SERMs), aromatase inhibitors, and gonadotropin-releasing hormone (GNRH)-analogs, is the standard treatment for hormone-receptor positive breast cancer [11]. There are new inhibitors with potential use in breast cancer therapy, such as poly(ADP-ribose) polymerase (PARP) inhibitors [12,13,14] or the inhibitors of CDK4/6 (cyclin-dependent kinases) [15].

For further information regarding the clinico-pathology of breast cancer, we refer the Readers to the relevant guidelines [1,16] and draw the attention of the Readers to use the most up-to-date version of the guidelines.

## 2. The Dysregulation of Metabolism in Breast Cancer

Breast cancer cells show characteristic pathological changes in metabolism and, in line with that, the pathological metabolism of the host (e.g., obesity, metabolic syndrome, type II diabetes) increases breast cancer risk that we discuss below briefly; for comprehensive reviews, see [17,18,19,20,21] and Table 1. 

Originally, Otto Warburg suggested that cancer cells switch for “aerobic glycolysis” that are characterized by increased glycolytic flux and decreased mitochondrial oxidation that supports the high proliferative capacity of cancer cells [22]. Indeed, breast cancer cells were shown to exert features of Warburg metabolism [23], and in line with that, reverting Warburg metabolism can slow down the proliferation of breast cancer cells or declutch cell death, too [24]. Later, besides changes to glycolysis and mitochondrial oxidation, other metabolic pathways were shown to be upregulated in breast cancer [25], such as glutamine metabolism [26], lipid and fatty acid [27,28,29,30,31], glutamine-serine pathway [26,32,33], protein translation [34], or cholesterol metabolism [21]. These changes are the consequences of a complex rearrangement of the cellular energy sensor web, such as the activation of hypoxia-inducible factors (HIFs) [35,36], mammalian target of rapamcyin (mTOR) [37,38,39,40], estrogen-related receptors [41], estrogen receptors [19], phosphatidyl-inositol-3 kinase (PI3 kinase) [42,43], AMP-activated protein kinase (AMPK) [44,45], peroxisome proliferator-activated receptor cofactor-1α and β (PGC1α and PGC1β) [46,47], or nuclear respiratory factor 1 (NRF1) [46].

Metabolic changes have important pathological roles, as these changes have important roles in supporting proliferation [24,48], angiogenesis [49], or epithelial-to-mesenchymal transition (EMT) [50,51]. It is also very likely that two risk factors of breast cancer, obesity or type II diabetes, at least in part, increase risk through predisposing for changes towards pathological metabolism in (cancer) cells [52,53,54,55,56,57]. Breast cancer metabolism is an attractive new target for chemotherapeutic interventions [19]; furthermore, changes to metabolism in breast cancer can be used to monitor the efficiency of chemotherapy [58] and changes to cancer cell metabolism can be exploited to overcome chemotherapy resistance [19,59,60].

There are four molecular (intrinsic) subtypes of breast cancer: luminal A (ER+, low proliferative capacity), luminal B (ER+, high proliferative capacity), HER2-enriched (ER−, HER2+), and basal-type (triple negative breast cancer, TNBC) [61]. All of the subtypes have metabolic alterations and there is compelling evidence that it is possible to discriminate between the intrinsic subtypes as a function of changes to metabolism [30] (see Table 1). These results also highlight the strong contribution of pathological metabolism of breast cancer cells to proliferative capacity and aggressiveness.

The discovery of differences between the metabolism of cell types of breast cancer makes the landscape of the metabolic changes more complex. Namely, cancer stroma cells (dubbed non-tumorigenic cancer cells) or cancer-associated fibroblasts indeed rely on Warburg metabolism; however, the metabolism of cancer stem cells is dominated by mitochondrial oxidation [49,60,79,80,81,82,83,84,85,86,87,88]. Cancer stem cells in breast cancer have two different forms, the mesenchymal-like cancer stem cells are CD44+/CD24−, while the epithelial-like cancer stem cells are aldehyde dehydrogenase 1 family, member A1 (ALDH1) positive [89,90]. The inhibition of mitochondrial oxidation can increase the proportions of cancer stroma to stem cells, facilitating the efficiency of conventional chemotherapy that primarily targets stromal cells [86,91,92,93]. Larger proportions of stem cells than ER+ positive cancers characterize TNBCs [82,83]. In support of that observation, the TCA cycle is more active in TNBC as compared to the ER+ cases [94,95]. There is accumulating evidence that the metabolism of circulating cancer cells change to a more oxidative phosphorylation-dependent metabolism [19].

The actual shape of cancer cell metabolism is sharply regulated by the tumor microenvironment: the abundance of collagen [96], the circulating cytokines [97], or the adipocytes surrounding the tumor [52] are all major determinants of cancer cell metabolism. In this review, we add a new component to this list, bacterial metabolites, which are produced by the gut microbiome.

## 3. Microbiome Dysbiosis in Breast Cancer

Dysbiosis denotes an abnormal composition or maladaptation of the microbial community (the microbiome) of a given organism or a given compartment of the organism. Dysbiosis disrupts the normal function of the microbial community through hampering the symbiotic relationships in the community (e.g., cross-feeding). Dysbiosis can occur when the composition or the bacterial biomass changes (i.e., the proportions of certain species change within the community) [98]. Dysbiosis characterizes several neoplastic diseases [99,100,101,102,103,104], including breast cancer [91,105,106,107,108,109,110,111,112,113,114,115,116,117,118,119,120,121,122,123,124]. Although the largest microbial community of the human body resides in the gastrointestinal tract, when discussing dysbiosis in breast cancer, the breast’s own microbiome also has to be taken into account. This, yet, ill-characterized, bacterial community that is found in the milk-ducts of the breast also exhibits dysbiosis in breast cancer. Studies have shown dysbiosis in the breast microbiome [105,106,107,108,109,110,111,112,113,114,115] (Table 2) and in the fecal microbiome [91,116,117,118,119,120,121,122,123] (Table 3).

Obtaining nipple aspirate fluid or sterile biopsy are starting materials for the analysis of the breast microbiome that is then subjected to next-generation sequencing. The biomass of the breast microbiome decreases in breast cancer patients [105]. One explanation for the variability could be that the geographical difference creates notable changes in the composition of the microbiome, as pointed out in [110]. Changes to the breast microbiome are in correlation with the molecular subtype (hormone receptor+, HER2+, or triple negative), where the microbiome composition of the triple negative cases differs from other types [107,113]. Furthermore, the breast microbiome change as a function of the grade [115] and aggressivity [109] of the disease. These changes are translated into functional changes, as in the breast microbiome glycerophospholipid biosynthesis and ribosome biosynthesis processes are upregulated, while the flavonoid biosynthesis decreased as the grade of the disease increases [115]. It is of note that there are also characteristic changes to the virome and fungome of the breast [113].

The fecal microbiome is also characterized by changes in breast cancer patients. The group of James J. Goedert published a series of studies illustrating that the diversity of the gut microbiome decreases in breast cancer patients as compared to healthy controls and the relative abundance of *Clostridiales* increase in patients [116,117,118,119]. Using the biobank set-up by the Goedert group [118], Miko and co-workers [120] and Kovacs and colleagues [91] assessed a subset of bacterial species and showed that the most drastic decreases were observed in early stage breast cancer (stage 0 and stage 1). These observations are supported be the observations of Luu and colleagues [121]; the log10 equivalent number/g stool of all *Bacteroidetes*, *Clostridium coccoides*, *Clostridium leptum*, *Faecalibacterium prausnitzii,* and *Blautia* species increased in stage II and III patients as compared to the stage 0 and I patients. In contrast to that, Zhu and co-workers [122] showed that the diversity of the gut microbiome changes differently in the pre- and postmenopausal breast cancer patients, furthermore the diversity of the gut microbiome increases in patients when compared to healthy controls. 

There are correlative studies showing that antibiotic consumption, which decreases the diversity of the microbiome, increases the risk and recurrence of breast cancer [126,127,128,129,130,131,132]. Despite the non-mechanistic nature of these studies and the chance for uncontrolled confounding, these studies strengthen the observations that the decreases in the diversity of the microbiome increase the risk for breast cancer. Importantly, a recent murine study [132] showed that the use of a cephalosporin antibiotic (Cephalexin) accentuated the decrease in microbiome diversity that was induced by the tumor itself and induced tumor formation, suggesting a causative relationship between antibiotic use and breast cancer incidence, as well as strengthening the hypothesis of the reduced bacterial diversity in breast cancer. Cephalexin reduced the abundance of *Odoribacter* and *Anaeotruncus* (both are butyrate-producing bacterial groups), while increasing the abundance of *Bacteroides* [132]. 

## 4. Interactions between Microbiome and Breast Cancer Cells—Metabolites in Action

**Figure 1:**
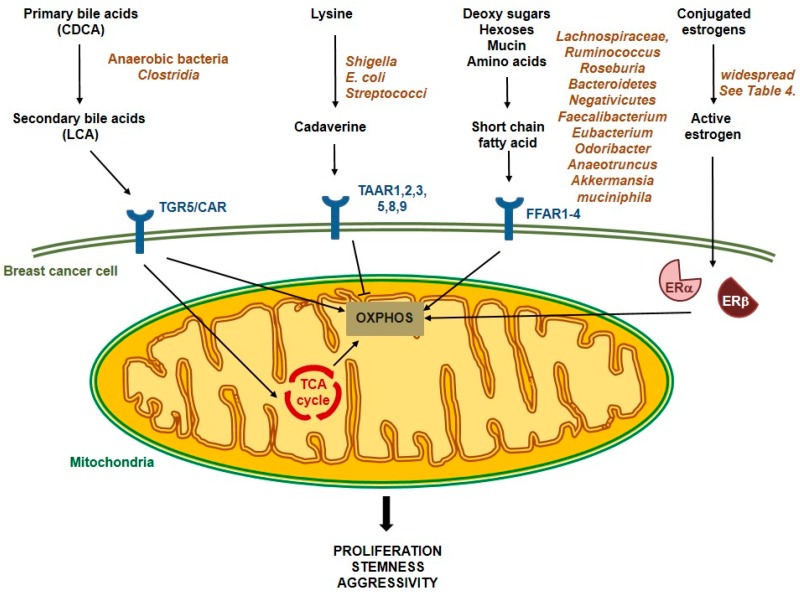
Schematic representation of the pathways elicited by bacterial metabolites that modulate mitochondrial metabolism in breast cancer.

There are multifaceted bidirectional interactions between the host and the microbiome [133]. The host regulates the composition of its microbiome through its innate immune system or its feeding or hygiene behavior, but, in turn, as recent research strongly argues, the microbiome can also fine tune the (patho)physiology of the host [133,134,135]. A major pathway in microbiome-to-host signaling is the secretion of bacterial metabolites that enter the circulation and reach their target cells [136,137,138,139]. In that respect, the function of these bacterial metabolites is similar to human hormones, which are synthesized in an organ or gland (in this case, it is the microbiome) and they are transferred to other anatomical locations, where they exert their biological effects. Such blood-borne microbial metabolites were shown to modulate the behavior of breast cancer, lithocholic acid (LCA) [120,140,141,142,143], short chain fatty acids (SCFA) [134,144], cadaverine [91], or deconjugated estrogens [116,117]. These bacterial metabolites have profound impact on mitochondrial metabolism, nevertheless it is of note that metabolites also regulate other metabolic processes (e.g., lipid metabolism) [21] (For overview, please see Table 4 and Figure 1).

### 4.1. Estrogen Deconjugation and Reuptake

The group of James J. Goedert showed that, in men and postmenopausal women, the gut microbiome is a key determinant of estrogen metabolism [116,117]. The microbiome has a vital role in estrogen metabolism, as bacteria can deconjugate excreted estrogens enabling their reuptake [168], in line with that, the urinary estrogen levels showed correlation with the richness of the fecal flora in men and in postmenopausal women [116,117]. Bacterial β-glucuronidases are responsible for the deconjugation of conjugated estrogens that are coded by the *gus* [145,146] and *BG* genes [147]. *gus* is widespread among gut bacteria, being more common among *Firmicutes*, while BG is more widespread, being present in *Bacteroidetes* and *Firmicutes* [147]. The following bacterial genuses were shown to express β-glucuronidases: *Collinsella, Edwardsiella, Alistipes, Bacteroides, Bifidobacterium, Citrobacter, Clostridium, Dermabacter, Escherichia, Faecalibacterium, Lactobacillus, Marvinbryantia, Propionibacterium, Roseburia,* and *Tannerella* [169]. Goedert and colleagues have provided strong functional evidence for the role of *Clostridiales* in estrogen reactivation [116,117] and showed that the relative abundance of *Clostridiales* increases in breast cancer patients [116,117,118]. Feeding regimes can modulate β-glucuronidase expression in the gut [146]. (Reactivated) estrogens act through estrogen receptors, and hence promote the progression of ER+ breast cancer through multiple pathways (Table 1).

Reactivation of estrogens enable their reuptake and increase serum estrogen levels [116,117,118], estrogen-evoked changes in the expression of mitochondrial genes were suggested to contribute to estrogen-induced carcinogenesis [170]. In line with that, both estrogen receptors (ER), ERα and ERβ, reside on the surface of mitochondria [171] and the ER-responsive sites were suggested to be present in mitochondrial DNA [170]. ERβ is directly involved in the expression of nuclear-coded mitochondrial proteins [172]. Endocrine-resistant tumors have higher mitochondrial respiration when compared to the tumors that are sensitive for endocrine therapy, which is due to the increased expression of NRF1 and TFAM1 [148,149]. Increased oxidative phosphorylation was shown to contribute to tamoxifen-resistance [150] and general therapy failure [173], support metastasis [19], and render the tumors more aggressive [151]. The flip-side of the induction of mitochondrial oxidation is the increased mitochondrial production of free radicals that is cytostatic and is dependent on ERβ [174,175]. When taken together, bacterial estrogen deconjugation can promote breast cancer progression.

### 4.2. Short-Chain Fatty Acid Production

The microbiome by the saccharolytic fermentation of non-digestible carbohydrates generate short-chain fatty acids (SCFAs, acetate, propionate, butyrate) [161,176]. Fermentation of the non-digestible carbohydrates yield formate, acetate, propionate, butyrate, and lactate [176]. A relatively small proportion of SCFAs are produced through amino acid degradation; the degradation of branched chain amino acids yield branched-chain fatty acids; nevertheless, the amount of branched-chain fatty acids is extremely low [176].

The capability for acetate production is widespread among bacteria, while the production of other metabolites is more restricted to certain species. *Akkermansia muciniphila* is a key player in propionate production from mucin [155], while *Lachnospiraceae*, *Ruminococcus obeum,* and *Roseburia inulinivorans* are responsible for the degradation of deoxy sugars (e.g., fucose, rhamnose) and hexoses by *Bacteroidetes* and *Negativicutes* sp. to produce propionate [157]. The majority of butyrate production is bound to *Faecalibacterium prausnitzii,* *Eubacterium rectale*, *Roseburia faecis*, *Eubacterium hallii,* and an unnamed cultured species SS2/1 [156], as well as the genera *Odoribacter* and *Anaeotruncus* [132]. It is also of note that the application of Cephalexin, which is an antibiotic that is frequently used as a pre-surgery premedication to breast cancer patients, reduced the abundance of *Odoribacter* and *Anaeotruncus* that are butyrate producer bacteria [132]. The abundance of *Akkermansia muciniphila,* which is a cross-feeder and propionate producer species, was associated with the richness of the gut microbiome in breast cancer patients [125].

The serum concentration of the total SCFAs fall into the 10–100 µM range, wherein acetate, propionate, isobutyrate, and butyrate make up the bulk [177,178,179]. SCFAs modulate numerous cancer hallmarks, such as cell proliferation, apoptosis, cell invasion, gene expression, metabolism, among others, in breast cancer [144,159,180,181,182]. The main receptors of SCFAs are the free fatty acid receptors (FFARs) that are only situated on the cancer cells, but also on stromal cells (e.g., adipocytes) [180,183,184]. The effects of SCFAs can have positive (e.g., [144]) and negative (e.g., [184]) effects in breast cancer as a function of the context.

The knowledge on role of SCFAs in mediating metabolism in breast cancer cells is very limited. In breast cancer cells, even-chain short fatty acids, acetate, butyrate, or lactate can be directly utilized as energy substrates, in line with that, sodium-butyrate induces oxygen consumption in breast cancer cell lines [158] and the inhibition of the lactate metabolism sharply reduces the viability of breast cancer cells [185]. Furthermore, butyrate can induce apoptosis through inducing mitochondrial ROS generation [159]. SCFAs, most notably, butyrate, are histone deacetylase inhibitors that are a key feature for their anticancer activity [160,161,162,163].

### 4.3. Secondary Bile Acid Metabolism

Lithocholic acid is secondary bile acid that is synthesized from chenodeoycholic acid (CDCA) and ursodeoxycholic acid (UDCA), by the dehydroxylation at position 7 [165,186]. The genes that are involved in the degradation of secondary bile acids can be found in the bile acid-inducible operon (bai operon) [164]. The enzyme catalyzing the formation of lithocholic acid that is cytostatic in breast cancer is 7α/β-hydroxysteroid dehydroxylase (*baiH*) [165,186]. Anaerobic bacteria, mostly the *Clostridiales,* are responsible for bile acid transformation [164]. The bile acids in the breast are of the gut origin [187].

The capacity of the human body and the microbiome to synthesize LCA is largely reduced in breast cancer, which is the most dominant in early stages (stages 0 and 1) [120]. Serum lithocholic acid levels negatively correlate with Ki67 labelling index in breast cancer [167]. LCA, in concentrations corresponding to its serum or breast tissue concentrations (30–50 nM or < 1 µM, respectively [120,188]), exerts antineoplastic effects on breast cancer cells by inhibiting epithelial-to-mesenchymal transition, vascular endothelial growth factor (VEGF) production, metastasis formation, induced antitumor immunity, and elicited changes in metabolism [120]. In supraphysiological concentrations (>1 µM), LCA inhibits fatty acid biosynthesis [143], induces induced multidrug resistance proteins [166], and induces cell death [140,141,143,166]. LCA did not exert antiproliferative effects in its tissue reference concentrations on non-transformed primary fibroblasts [120]. A exert its antineoplastic effects through the G protein-coupled bile acid receptor 1 (TGR5) [120] and in supraphysiological concentrations through Farnesoid X receptor (FRX) [166]. Other secondary bile acids, deoxycholic acid (DCA) or ursodeoxycholic acid (UDCA), had no effect on breast cancer cells in the reference concentration [120].

Bile acid-induced activation of TGR5 was shown to induce OXPHOS in metabolic models [189,190,191,192], in good accordance with that, LCA can elicit anti-Warburg effects in breast cancer models. In breast cancer cells, LCA induces mitochondrial biogenesis through NRF1, AMPK, and PGC-1β; the same effectors are also induced in murine breast cancer models upon LCA feeding [120]. The induction of these energy sensors declutch transcription programs that induced the expression of mitochondrial proteins (cytochrome c, atp5g1, ndufb5) and consequently enhanced mitochondrial activity. both when, the TCA cycle was fed on acetate or on glucose suggesting enhanced glycolytic flux, too [120]. In parallel, oxygen consumption rates were also induced suggesting improved terminal oxidation [120]. Besides the regulation of oxidative phosphorylation, LCA induced mesenchymal-to-epithelial transition, antitumor immune response, and inhibited proliferation and metastasis formation [120] (see Table 1).

### 4.4. Amino Acid Degradation

Cadaverine is synthesized from lysine by the bacterial enzymes LdcC and CadA [193,194]. Human cells are also capable of synthesizing cadaverine; however, it seems that bacterial cadaverine production is dominant over human biosynthesis [91]. *Shigella flexneri*, *Shigella sonnei*, *Escherichia coli*, and *Streptococci* were shown to express cadaverine biosynthetic enzymes [195].

Cadaverine in concentrations corresponding to its serum reference concentrations (100–800 nM) [196,197] inhibited cell proliferation, epithelial-to-mesenchymal transition, cell movement and invasion, and tumor infiltration to the surrounding tissues [91]. Moreover, cadaverine changed metabolism in breast cancer cells and it reduced the proportion of ALDH1^+^ cancer stem cells in 4T1 murine breast cancer cells [91]. Cadaverine exerted its effects through the trace amine-associated receptor-1, 2, 3, 5, 8, 9 (TAAR1, 2, 3, 5, 8, 9), of which TAAR1 was already associated with the inhibition of breast cancer growth [198]. The capacity of the microbiome to synthesize cadaverine is suppressed in breast cancer, most dominantly in early stage breast cancer (stages 0 and 1) [91]. Putrescine had no effect on breast cancer cells [91]; furthermore, cadaverine was not effective on primary, untransformed cells [91].

Cadaverine has been shown to reduce cellular oxygen consumption that is a readout of OXPHOS activity, rendering the cells more glycolytic [91]. The molecular mechanisms bringing about that phenotype has not yet been elucidated. In line with the more glycolytic phenotype of cells, the percentage of cancer stem cells was reduced upon cadaverine treatment [91]. In addition to these, cadaverine inhibited migration, invasion, and metastasis formation, as well as induced mesenchymal-to-epithelial transition [91].

## 5. Interference of the Microbiome and Anticancer Treatment

Bacteria of the microbiome can interfere with chemo- and radiotherapy in cancer treatment and management [199,200], which is also true for breast cancer. There are several aspects to the interactions between the microbiome and anticancer treatment; the microbiome can metabolize the chemotherapeutic drugs, inactivating or activating them, can modulate the immune system [201,202], can interfere with the side-effects of therapy, or the therapy can modulate the microbiome. Alexander and colleagues [203] suggested a framework, called TIMER (from Translocation, Immunomodulation, Metabolism, Enzymatic degradation, and Reduced diversity and ecological variation), to support a coordinated description of the interactions between the microbiome and cancer drugs. Below, we discuss the drugs that are relevant for breast cancer treatment.

Anthracyclines are synthesized by *Streptomyces* strains and can hence modulate the composition of the microbiome [204], for example, anthracyclines can be bacteriostatic on *Acinetobacter* species [205]. Several bacteria can metabolize anthracyclines (i.e., detoxify them) [206,207,208]; *Streptomyces* WAC04685 can deglycosylate and inactivate doxorubicin [209]. *Streptomycetes* can be found with a low prevalence in the human gut [210], making it likely that the microbiome can interfere with the bioavailability and pharmacokinetics and the pharmacodynamics of anthracyclines. Furthermore, upon anthracycline treatment, certain bacteria can cross the intestinal barrier to enter secondary lymphoid organs [203].

Cyclophosphamides cause damage to the gut mucosa and, thereby, attenuate the barrier function that makes the gut leaky and gut bacteria can enter [211]. Rich microbiome is protective against cyclophospmamide-induced mucosal injury [212]; in fact, strains of *Lactobacillus plantarum* as a probiotic was shown to be protective against mucosal injury [213]. Bacteria (usually Gram-positive microorganisms, such as *Lactobacillus johnsonii*, *L. murinus,* *Barnesiella intestinihominis*, and *Enterococcus hirae* [203,214]) can enter secondary lymphoid organs and thereby shape the anticancer immune response of the host [202]. *Lactobacillus plantarum* HY7712 can ameliorate cyclophosphamide-induced immunosuppression in mice [215].

Selective estrogen receptor modulators (SERMs) (Tamoxifen, Raloxifen) tamoxifen can modulate the composition of the microbiome. SERMs can be toxic for *Pseudomonas aeruginosa*, *Klebsiella pneumoniae*, *Acinetobacter* *baumannii* [216,217,218], *Porphyromonas gingivalis*, *Streptococcus mutans* [219], *Enterococcus faecium* [220], and *Bacillus stearothermophilus* [221]. To date, no bacterial drug metabolism was related to SERMs. Tamoxifen resistance was shown to be a reason of changes to cancer cell metabolism [63,149,222], which can be modulated by the microbiome.

Taxanes can be a subject to bacterial metabolism [223,224]. Moreover, taxanes may interfere with bacterial LPS in activating the immune system [225]. Finally, taxanes can interfere with the composition of the microbiome [226].

Antimetabolites, 5-fluorouracil (5FU), and gemcitabine were shown to interact with the microbiome. Both of the drugs are metabolized by the microbiome [227,228,229,230,231,232,233]. Bacterial enzymes can activate both drugs [203,233,234]; nevertheless, the bacterial deactivation of the active metabolites is equally important [203,229,230,231]. Although, studies in breast cancer or its models had not been performed, it is known that the bacterial metabolism of 5FU can influence the treatment efficacy of colorectal cancer [235]. Intratumoral bacteria (*Gammaproteobacteria*) were shown to be the key players in deactivating gemcitabine in colorectal cancer that was alleviated by the eradication of *Gammaproteobacteria* by ciprofloxacin [229]. A loss of gemcitabine cytotoxicity was also observed in Mycoplasma-infected cells in culture [230]. Geller and Straussman [232] identified *Gammaproteobacteria* in human pancreatic ductal adenocarcinoma tumors in elevated numbers than in healthy pancreas tissue that can regulate gemcitabine availability. This observation can be extended for breast cancer, as it is also a solid tumor inside the human body. Cytidine deaminase was shown to be responsible for gemcitabine inactivation [232]. Both 5FU and gemcitabine have bactericide properties [231,234,236,237] and, therefore, they modulate the composition of the microbiome. Capecitabine was not toxic on *E. coli* [231] and its effects on the microbiome is largely uncharacterized. 5FU-induced dysbiosis contributes to the severity of the 5FU-induced mucositis [238,239] that can be corrected by the use of probiotics [240]. 5FU mucositis can induce bacterial translocation through the intestinal barrier [241].

PARP inhibitors, which are drugs likely to be used in the future in breast cancer treatment [12,13,14], were shown to increase the diversity of the gut microbiome [242,243]. To date, there is no literature on the interplay between aromatase inhibitors, navelbine, GNRH-analogs, and the microbiome. It is of note that bacteria can also interfere with biological therapies; nevertheless, no interactions were found with the antibodies used in breast cancer targeted therapy [203]. Finally, the microbiome can modulate the severity of radiation-induced mucositis [244,245] and can protect against radiation-induced toxicity [246]. 

## 6. Applicability and Future Directions

Bacterial dysbiosis characterizes breast cancer, both in the breast tissue and in the gut [91,105,106,107,108,109,110,111,112,113,114,115,116,117,118,119,120,121,122,123,247]. Both the gut and breast microbiome sharply responds to the disease and display changes as a function of the histological variants, grade, or stage of the disease [91,107,109,113,115,120,121]. The role of the breast microbiome in carcinogenesis is unresolved, in contrast to that, the gut microbiome was shown to produce or modify metabolites (e.g., LCA, cadaverine, SCFAs, estrogens), which, through the circulation, get to distant sites, such as the breast, where they modulate cancer cell function. In that sense, these metabolites resemble human hormones, as they are produced in a “gland” (in this case, the microbiome) and are subsequently transferred to distant sites of action through the circulation. These metabolites appear to be important constituents of the tumor microenvironment. 

All known bacterial metabolites have pleiotropic effects on breast cancer cells; nevertheless, almost all of them modulate mitochondrial metabolism. However, the actual effects are rather variable and there are inducers (e.g., estrogens or LCA) and inhibitors (e.g., cadaverine) of mitochondrial metabolism. Changes to mitochondrial metabolism is a double-edged sword in breast cancer. Switching for an anti-Warburg mitochondrial metabolism, comprising the upregulation of mitochondrial oxidation, can slow the proliferation of cancer cells and sensitize stromal cell for chemotherapy; however, in parallel, the induction of mitochondrial oxidation can tune cancer cells into cancer stem cells [85,86,93,248,249]. An example for that is cadaverine, which represses mitochondrial oxidation and, hence, reduces the percentage of cancer stem cells [91]. It is also of note that the substrate availability and substrate preference of cancer cells can also drive a switch between cancer stroma and cancer stem cells [250,251,252]. Consequently, a good understanding of metabolite-induced changes can enable us to use these metabolites in combatting breast cancer, either as stand-alone drugs or in combination with other chemotherapy regimens or mitochondrial drugs.

There is an intricate connection with feeding regimes and breast cancer risk [253], suggesting that it may be possible to create feeding regimes that maintain a “cytostatic microbiome” [134,135]—probiotics and changes to diet can influence SCFA serum levels [134]. Finally, there are natural dietary compounds (e.g., polyphenols) that can also modulate the microbiome and mitochondrial metabolism [254]. Maintaining the microbiome in good shape can be also vital in the successful completion of chemotherapeutic regime in breast cancer treatment [238,239,240]. 

The microbiome may also have indirect effects on breast cancer. For example, the white adipose tissue has aromatase activity and, therefore, it can synthesize estrogen and thereby promote breast cancer. Pathological composition of the microbiome is also associated with obesity [255] and increases in the body mass index (BMI) are a risk factor of breast cancer [125,256]. There are other drivers of dysbiosis, apart from antibiotics or obesity, such as aging [257,258,259,260] or diseases (e.g., type II diabetes, polycystic ovary syndrome, non-alcoholic fatty liver disease, etc. [261,262,263,264,265,266,267]), which may explain the association of cancer events with these diseases. Bacteria in the microbiome can serve as sources of immunogenicity, similarly to the appearance of the counter-antibodies of the AB0 blood group system [268,269], and through that could modulate the activity of the immune system [202,214,270,271,272,273]. Finally, cachexia is also associated with dysbiosis [274,275,276,277].

We are still scratching the surface in understanding the role of dysbiosis in breast cancer. Nevertheless, there seems to be a meaningful, complex, and deep molecular network, below which can be exploited in the combat against cancer.

## Figures and Tables

**Table 1:** Metabolic changes in the intrinsic subtypes of breast cancer. Empty squares stand for no data. Abbreviations: ASCT2/SLC1A5, amino acid transporter-2; ER, estrogen receptor; GDH/H6PD, glutamate dehydrogenase; GLS1, glutaminase 1; HER2, human epidermal growth factor 2 receptor; PgR, progesterone receptor; SLC, solute carrier transporters.

	Breast Cancer	Luminal A	Luminal B		HER2+	TNBC (~Basal-like)	Ref.
			HER2−	HER2+			
Receptor Status according to [1]	N/A	ER+, HER2−, Ki67 ^low^, PgR ^high^ Low-Risk Molecular Signature (If Available)	ER+, HER2−, either Ki67^high^ or PgR ^low^ High-Risk Molecular Signature (If Available)	ER+, HER2+, any Ki67, any PgR	HER2+, ER−, and PgR−	HER2-, ER-, and PgR−	[1]
Cholesterol and oxysterol metabolism	Lipid and cholesterol metabolism supports tamoxifen resistance. Increases in serum cholesterol is a risk factor for breast cancer.	27-hydroxycholesterol supports the growth of ER+ breast cancer cells	27-hydroxycholesterol supports the growth of ER+ breast cancer cells	27-hydroxycholesterol supports the growth of ER+ breast cancer cells			[62,63,64,65,66,67,68]
Glycolysis	upregulated	low	intermediate/low	intermediate/low	intermediate/low	high	[69,70,71,72]
Pentose-phosphate pathway	upregulated	low	low	low	high	highest	[73]
Glutamine-proline-glycine metabolism	upregulated to serve energy homeostasis and protein and nucleotide biosynthesis	SLC6A14, SLC7A11 upregulated	High expression of glutamine-proline enzymes in Myc^high^ tumors SLC6A14, SLC7A11 upregulated	High expression of glutamine-proline enzymes in Myc ^high^ tumors SLC6A14, SLC7A11 upregulated	highest expression of GLS1, GDH, ASCT, SLC7A5, SLC1A5 upregulated highest level of glutamine metabolism among the intrinsic types	SLC7A11, SLC1A5 upregulated increased glutamine uptake	[37,38,39,74,75]
Protein translation	upregulated	highest	high	high			[76,77,78]

**Table 2:** Studies investigating changes to the breast microbiome in breast cancer. Abbreviations: ER, estrogen receptor; CNB, Core needle biopsies; HER2, herceptin receptor/erbB receptor; NAF, Nipple aspirate fluid; PR, progesterone receptor; SEB, Surgical excision biopsies; TNBC, Triple negative breast cancer.

Sample Type and Sample Size	Method	Observations	Changes to the Microbiome	Ref.
Breast tumor tissue and paired normal adjacent tissue from the same 20 patient (ER positive)	Pyrosequencing 16S V4 rDNA	The amount of bacteria, measured by the copy number of 16S rDNA, is not significantly different in paired normal tissue from breast cancer patients and healthy breast tissue from healthy individuals. The amount of bacteria, measured by the copy number of 16S rDNA, is significantly reduced in breast cancer tissue.	The most abundant phyla in breast tissue were *Proteobacteria*, *Firmicutes*, *Actinobacteria* and *Bacteroidetes*. *Methylobacterium radiotolerans* is relatively enriched in tumor tissue and *Sphingomonas yanoikuyae* is relatively enriched in paired normal tissue.	[105]
Breast tissue from 81 women with and without breast cancer from Canada and Ireland. Canadian patients: benign (*n* = 11), cancerous tumors (*n* = 27) and healthy individuals (*n* = 5) Irish patients: breast cancer (*n* = 33) and healthy individuals (*n* = 5)	Ion Torrent V6 16S rRNA sequencing and culture	Breast tissue contains a diverse population of bacteria. Geographical difference exist between breast tissue microbiome of Canadian and Irish subjects.	*Proteobacteria* and *Firmicutes* (specifically the class *Bacilli*) were the most abundant phyla in breast tissue. The most abundant taxa in the Canadian samples were: *Bacillus* (11.4%), *Acinetobacter* (10.0%), *Enterobacteriaceae* (8.3%), *Pseudomonas* (6.5%), *Staphylococcus* (6.5%), *Propionibacterium* (5.8%), *Comamonadaceae* (5.7%), *Gammaproteobacteria* (5.0%), and *Prevotella* (5.0%). The most abundant taxa in the Irish samples were: *Enterobacteriaceae* (30.8%), *Staphylococcus* (12.7%), *Listeria welshimeri* (12.1%), *Propionibacterium* (10.1%), and *Pseudomonas* (5.3%). Higher abundance of *Escherichia coli* was detected in women with cancer than in healthy controls.	[110]
Triple negative breast cancer (TNBC) samples (*n* = 100)	PathoChip array	There are unique microbial signatures in triple negative breast cancer.	Multiple viruses and other microorganisms were detected in triple negative breast cancer samples. Bacterial signatures: *Brevundimonas diminuta*, *Arcanobacterium haemolyticum*, *Peptoniphilus indolicus*, *Prevotella nigrescens*, *Propiniobacterium jensenii* and *Capnocytophaga canimorsus* (see in [107])	[107]
Nipple aspirate fluid (NAF) from healthy women (*n* = 23) and from women with breast cancer (*n* = 25)	16S V4 rRNA gene sequencing	Microbiome composition of NAF from healthy control and breast cancer are significantly different. Beta-glucuronidase levels are higher in NAF from breast cancer than from healthy control.	The most abundant phyla in NAF were *Firmicutes*, *Proteobacteria*, and *Bacteroidetes*. In NAF from breast cancer genus *Alistipes* was more abundant and an unclassified genus from the *Sphingomonadaceae* family in NAF from healthy women.	[108]
Breast tissues from patients with benign (*n* = 13) and invasive breast cancer (*n* = 15). The invasive cancers were stage I in 10 patients (67%) and stage II in 5 (33%). Tumors were histologic grade I in 43% and grade II in 57%. The invasive cancers were all ER and PR positive, and a minority (29%) were HER2 positive.	16S V3-V5 rDNA hypervariable taq sequencing	Breast tissue microbiome is different in women with malignant disease and in women with benign disease.	The most abundant phyla in breast tissue were *Firmicutes*, *Actinobacteria*, *Bacteroidetes* and *Proteobacteria*. Breast cancer malignancy correlated with enrichment in taxa of lower abundance including the genus *Fusobacterium*, *Atopobium*, *Gluconacetobacter*, *Hydrogenophaga*, and *Lactobacillus*.	[109]
Breast tissue from 58 women: benign (*n* = 13), cancerous tumors (*n* = 45), and healthy individuals (*n* = 23)	16S V6 rRNA sequencing	Different microbiome profile exist between breast tissue from healthy women and women with breast cancer. Normal tissues from women with benign tumors are more similar to normal adjacent tissues from cancer patients than to normal tissues from healthy women.	Breast cancer patients had higher relative abundances of *Bacillus*, *Enterobacteriaceae* and *Staphylococcus*. *Lactococccus* and *Streptococcus* were higher in healthy women than in breast cancer patients.	[106]
Breast tissue from 39 breast cancer patients (*n* = 17 tumor, *n* = 22 normal) and breast tissue from 24 healthy patients	16S V3-V4 rRNA sequencing	Microbiome of tumor and paired normal tissues from the same breast cancer patient are similar. Breast tissue from cancer and non-cancer patients have significantly different microbiome.	Decreased relative abundance in the genus *Methylobacterium* (phylum *Proteobacteria*) was found in breast cancer patients.	[111]
Breast tissue from tumor (*n* = 668) and normal adjacent tissue (*n* = 72) from The Cancer Genome Atlas (TCGA)	16S V3-V5 RNA sequencing data	The microbial composition is associated with alterations in the host expression profiles.	The most abundant phyla in breast tissues are *Proteobacteria*, *Actinobacteria*, and *Firmicutes*. *Proteobacteria* was increased in the tumor tissues and *Actinobacteria* abundance increased in non-cancerous adjacent tissues. *Mycobacterium fortuitum* and *Mycobacterium phlei* are species differentially abundant in the tumor samples. Geneset enrichment suggested that *Listeria* spp was associated with the expression profiles of genes involved with epithelial to mesenchymal transitions. *H. influenza* was associated with the proliferative pathways: G2M checkpoint, E2F transcription factors, and mitotic spindle assembly.	[112]
Breast cancer tissues [ER or PR positive (*n* = 50), HER2 positive (*n* = 34), triple positive (*n* = 24), triple negative (*n* = 40)] and breast tissue from healthy individuals (*n* = 20)	PathoChip array	There are unique viral, bacterial, fungal and parasitic signatures in each breast cancer type. Triple negative and triple positive samples showed distinct microbial signature, while the ER positive and HER2 positive samples shared similar microbial pattern.	Unique and common microbial signatures in the major breast cancer types are summarized in Table 1 in [113] All four breast cancer types had dominant signatures for *Proteobacteria* followed by *Firmicutes*. *Actinomyces* signatures was also detected in each breast cancer types.	[113]
Fresh tissue samples of both cancer and paired healthy tissues from core needle biopsies (CNB; *n* = 12) and surgical excision biopsies (SEB; *n* = 7). 3 patients underwent both procedures	hypervariable region of the 16S-rRNA gene (V3)	More similarities than differences exist between tumors and adjacent normal tissues from CNB and SEB specimens. There are more differences between subjects than between healthy and cancerous tissues collected from the same patient.	In breast tissue *Proteobacteria* are the most abundant phylum followed by *Firmicutes*, *Actinobacteria* and *Bacteroidetes*. Presence of genus *Ralstonia* is associated with breast tissue. The relative abundance of *Methylobacterium* was different in certain patients.	[114]
Breast tissue from benign (*n* = 22) and malignant (*n* = 72) breast cancer patients (Chinese cohorts)	16S V1-V2 rRNA sequencing	Microbiome profile is different in benign and malignant diseases. Microbiome composition is different in histological grades of malignant breast tissue. There is a specific correlation of microbial biomarkers and microbial pathways with advanced disease. Glycerophospholipid metabolism and ribosome biogenesis pathways were upregulated in grade III tumor compared to grade I and II. Flavonoid biosynthesis was significantly lower in grade III compared to grade I and II.	The enriched microbial biomarkers in malignant tissue included genus *Propionicimonas* and families *Micrococcaceae*, *Caulobacteraceae*, *Rhodobacteraceae*, *Nocardioidaceae*, and *Methylobacteriaceae*. The relative abundance of family *Bacteroidaceae* decreased and the relative abundance of genus *Agrococcus* (family *Microbacteriaceae*) increased with the development of malignancy. Genus *Propionicimonas* and five families *Micrococcaceae*, *Caulobacteraceae*, *Rhodobacteraceae*, *Nocardioidaceae* and *Methylobacteriaceae* were abundant in malignant disease compared to benign disease.	[115]

**Table 3:** Studies investigating changes to the gut microbiome in breast cancer. Abbreviations: AM, Akkermansia muciniphila; bai, bile acid inducible operon (wherein the *baiH* ORF codes for 7-HSDH, a key enzyme in lithocholic acid biosynthesis); BMI, body mass index; CadA, acid-inducible lysine decarboxylase; ER, estrogen receptor; HAM, high AM relative abundance; HER2, herceptin receptor/erbB receptor; LAM, low AM relative abundance; LdcC, constitutive lysine decarboxylase; PR, progesterone receptor.

Sample Type and Sample Size	Method	Observations	Changes to the Microbiome	Ref.
Urine and fecal samples from men (*n* = 25), postmenopausal women (*n* = 7), and premenopausal women (*n* = 19)	Pyrosequencing of the V1-V2 region of 16S rRNA genes	The richness of the fecal microbiome was directly associated with systemic estrogens.	Non-ovarian systemic estrogens were significantly associated with fecal *Clostridia* taxa, including non-Clostridiales and three genera in the *Ruminococcaceae* family.	[116]
Urine and fecal samples from healthy postmenopausal women (*n* = 60)	Pyrosequencing of the V1-V2 region of 16S rRNA genes	Diversity of the gut microbiome were associated with patterns of estrogen metabolism.	Relative abundances of a number of taxa in the class *Clostridia* were directly associated with the ratio of estrogen metabolites to parent estrogen, while the genus *Bacteroides* was inversely associated with this ratio.	[117]
Urine and fecal samples from postmenopausal women with breast cancer (*n* = 48) and paired control women (*n* = 48)	Illumina sequencing and taxonomy	Postmenopausal women with breast cancer have altered fecal microbiota composition but estrogen-independent low diversity of gut microbiota.	Breast cancer patients had higher levels of *Clostridiaceae*, *Faecalibacterium*, and *Ruminococcaceae*; and they had lower levels of *Dorea* and *Lachnospiraceae*.	[118]
Fecal samples from breast cancer patients (*n* = 31). Clinical stages were stage 0 (*n* = 15), stage I (*n* = 7), stage II (*n* = 7), stage III (*n* = 2). Patients were ER positive/ PR positive (90%) and HER2+ (15%). 23 patients had a normal BMI and 8 were overweight	qPCR targeting 16S rRNA sequences	Microbiome composition in patients differ according to clinical characteristics and BMI.	In overweight patients, the number of total *Firmicutes*, *Faecalibacterium prausnitzii*, *Blautia* sp., and *Eggerthella lenta* bacteria was significantly lower than in the normal BMI patients. Total number of *Bacteroidetes*, *Clostridium coccoides* cluster, *Clostridium leptum* cluster, *Faecalibacterium prausnitzii*, and *Blautia* sp. were significantly higher in clinical stage II/III than in clinical stages 0/I. *Blautia* sp. is associated with a major histoprognostic grade.	[121]
Urine and fecal samples from postmenopausal women with breast cancer (*n* = 48) Clinical stages were in situ (*n* = 11), stage 1 (*n* = 25), stage 2 (*n* = 10), stage 3 (*n* = 2); 88% ER-positive and paired control women (*n* = 48)	16S V4 rRNA gene sequencing	Breast cancer patients have significant estrogen-independent associations with the IgA-positive and IgA-negative gut microbiota.	Breast cancer patients had significantly reduced alpha diversity and altered composition of both IgA-positive and IgA-negative fecal microbiota.	[119]
Fecal samples from premenopausal breast cancer patients (*n* = 18), premenopausal healthy control (*n* = 25), postmenopausal breast cancer patients (*n* = 44), postmenopausal healthy control (*n* = 46).	Illumina sequencing	Composition of gut microbiome differ between postmenopausal breast cancer patients and healthy controls while did not differ significantly between premenopausal breast cancer patients and premenopausal controls.	Enriched species in postmenopausal breast cancer patients were *Escherichia coli*, *Citrobacter koseri*, *Acinetobacter radioresistens*, *Enterococcus gallinarum*, *Shewanella putrefaciens*, *Erwinia amylovora*, *Actinomyces* sp. HPA0247, *Salmonella enterica*, and *Fusobacterium nucleatum*. *Eubacterium eligens* and *Roseburia inulinivorans* were less abundant species in postmenopausal breast cancer patients.	[122]
Fecal DNA samples from postmenopausal women with breast cancer (*n* = 48) and healthy women (*n* = 48) The original patient cohort is published in [118].	qPCR (primers were designed for the known baiH ORF in different bacteria)	Abundance of baiH ORF in bacterial species was different in breast cancer patients compared to healthy control women.	The abundance of baiH of *Clostridium sordelli*, *Pseudomas putida* and *Staphyloccoccus aureus* was lower in breast cancer patients. A more pronounced decrease in the abundance of the baiH of *Bacteroides thetaiotaomicron* and *Pseudomonas putida* were detected in early stage breast cancer patients.	[120]
Fecal samples from women with stage 0 to II breast cancer (*n* = 32)/presurgical weight- loss trial	16S V4 rRNA gene sequencing	Body composition of early stage breast cancer women is associated with Akkermansia muciniphila (AM), microbiome diversity and interleukin-6 level.	Relative abundance of AM was lower in women with higher body fat. Alpha diversity was higher in women with HAM. Higher *Prevotella* and *Lactobacillus* while lower *Clostridium*, *Campylobacter*, and *Helicobacter* genera were detected in HAM vs. LAM patients. IL-6 was associated with species richness and body composition, but not AM.	[125]
Fecal DNA samples from postmenopausal women with breast cancer (*n* = 48) and healthy women (*n* = 48) The original patient cohort is published in [118].	qPCR (primers were designed for known CadA and LdcC genes in different bacteria)	Abundance of the DNA coding LdcC and CadA in bacterial species was different in breast cancer patients compared to healthy control women.	The abundance of *Escherichia coli* CadA and also *Escherichia coli, Enterobacter cloacae* and *Hafnia alvei* LdcC DNA slightly decreased in breast cancer patients. Decreased CadA and LdcC abundance was more pronounced in clinical stage 0 patients as compared to the pool of all patients. In the feces of stage 1 patients *Escherichia coli* LdcC protein levels were markedly lower than in the healthy women.	[91]

**Table 4:** Effects of the bioactive bacterial metabolites in breast cancer. Processes in green are upregulated by the metabolite, in red those, that are downregulated. Black text stands for ambiguous data. Abbreviations: ER–estrogen receptor; FFAR–free fatty acid receptor; TGR5/GPBAR1–G protein-coupled bile acid receptor 1; FXR–farnesyl X receptor; TAAR–trace amine-related receptor; OXPHOS–oxidative phosphorylation; EMT–epithelial-to-mesenchymal transition; HDAC–histone deacetylase; CSC–cancer stem cell; VEGF–vascular endothelial growth factor.

Metabolite	Receptor	Bacteria	Ref.	Bacterial Enzyme	Neoplastic Processes	Ref.
**Reactivated estrogen**	ERα ERβ	*Firmicutes* *Collinsella* *Edwardsiella* *Alistipes* *Bacteroides* *Bifidobacterium* *Citrobacter* *Clostridium* *Dermabacter* *Escherichia* *Faecalibacterium* *Lactobacillus* *Marvinbryantia* *Propionibacterium* *Roseburia* *Tannerella*	[116,117,118,145,146,147]	β-glucuronidase (*gus*/BC)	OXPHOS tamoxifen resistance metastasis, aggressivity hormone-induced apoptosis EMT proliferation, metastasis	[148,149] [150] [19,151] [152] [153,154] [21]
**Short chain fatty acids** Acetate Propionate Butyrate Lactate	FFARs	*Akkermansia muciniphila* *Lachnospiraceae* *Ruminococcus obeum* *Roseburia inulinivorans* *Bacteroidetes* *Negativicutes* sp. *Faecalibacterium* *prausnitzii* *Eubacterium rectale* *Roseburia faecis* *Eubacterium hallii* SS2/1 *Odoribacter* *Anaeotruncus*	[132,155,156,157]	diverse	OXPHOS (direct energy substrates) apoptosis HDAC inhibition macrophage antimicrobial activity	[158] [159] [160,161,162,163] [163]
**Secondary bile acids** Lithocholic acid	TGR5 FXR	*Clostridiales*	[164,165]	7α/β-hydroxysteroid dehydroxylase (*baiH*)	apoptosis (in supraphyisiological conc.) proliferation VEGF production OXPHOS antitumor immunity EMT fatty acid biosynthesis movement, metastasis formation	[140,141,143,166] [120,167] [120] [120] [120] [120] [143] [120]
**Amino acid degradation** Cadaverine	TAAR1, 2, 3, 5, 8, 9	*Shigella flexneri* *Shigella sonnei* *Escherichia coli* *Streptococci*	[132,155,156,157]	Lysine decarboxylase (*LdcC*, *CadA*)	OXPHOS CSC movement, invasion EMT metastasis formation	[91] [91] [91] [91] [91]

## References

[1] Senkus E., Kyriakides S., Ohno S., Penault-Llorca F., Poortmans P., Rutgers E., Zackrisson S., Cardoso F. (2015). Primary breast cancer: ESMO Clinical Practice Guidelines for diagnosis, treatment and follow-up. Ann. Oncol..

[2] Breastcancer.org U.S. Breast Cancer Statisticshttps. www.breastcancer.org/symptoms/understand_bc/statistics.

[3] UK C.R. UK Breast Cancer Statistics. www.cancerresearchuk.org/health-professional/cancer-statistics/statistics-by-cancer-type/breast-cancer#heading-Zero.

[4] Bleyer A., Welch H.G. (2012). Effect of three decades of screening mammography on breast-cancer incidence. N. Engl. J. Med..

[5] Valencia O.M., Samuel S.E., Viscusi R.K., Riall T.S., Neumayer L.A., Aziz H. (2017). The Role of Genetic Testing in Patients With Breast Cancer: A Review. JAMA Surg..

[6] Minicozzi P., Van Eycken L., Molinie F., Innos K., Guevara M., Marcos-Gragera R., Castro C., Rapiti E., Katalinic A., Torrella A. (2019). Torrella AComorbidities, age and period of diagnosis influence treatment and outcomes in early breast cancer. Int. J. Cancer.

[7] Forrai G., Toth Z., Sebo E., Toth J. (2017). Theoretical and Practical Handbook of Assistants Working at Breast Screening Units (EMLŐDIAGNOSZTIKAI ASSZISZTENSEK ELMÉLETI ÉS GYAKORLATI KÉZIKÖNYVE).

[8] Kalager M., Zelen M., Langmark F., Adami H.O. (2010). Effect of screening mammography on breast-cancer mortality in Norway. N. Engl. J. Med..

[9] Warner E. (2011). Clinical practice. Breast-cancer screening. N. Engl. J. Med..

[10] Lauby-Secretan B., Scoccianti C., Loomis D., Benbrahim-Tallaa L., Bouvard V., Bianchini F., Straif K. (2015). Breast-cancer screening—Viewpoint of the IARC Working Group. N. Engl. J. Med..

[11] Harbeck N., Gnant M. (2017). Breast cancer. Lancet.

[12] Curtin N., Szabo C. (2013). Therapeutic Applications of PARP Inhibitors: Anticancer Therapy and Beyond. Mol. Aspects Med..

[13] Bai P. (2015). Biology of Poly(ADP-Ribose) Polymerases: The Factotums of Cell Maintenance. Mol. Cell.

[14] Fong P.C., Boss D.S., Yap T.A., Tutt A., Wu P., Mergui-Roelvink M., Mortimer P., Swaisland H., Lau A., O’Connor M.J. (2009). Inhibition of Poly(ADP-Ribose) Polymerase in Tumors from BRCA Mutation Carriers. N. Engl. J. Med..

[15] Kwapisz D. (2017). Cyclin-dependent kinase 4/6 inhibitors in breast cancer: Palbociclib, ribociclib, and abemaciclib. Breast Cancer Res. Treat..

[16] Badve S.S. (2018). Breast in AJCC Cancer Staging Manual, Eighth Edition.

[17] Ogrodzinski M.P., Bernard J.J., Lunt S.Y. (2017). Deciphering metabolic rewiring in breast cancer subtypes. Transl. Res..

[18] Tan J., Le A. (2018). Breast Cancer Metabolism. Adv. Exp. Med. Biol..

[19] Gandhi N., Das G.M. (2019). Metabolic Reprogramming in Breast Cancer and Its Therapeutic Implications. Cells.

[20] Martinez-Outschoorn U.E., Peiris-Pages M., Pestell R.G., Sotgia F., Lisanti M.P. (2017). Cancer metabolism: A therapeutic perspective. Nat. Rev. Clin. Oncol..

[21] Kulkoyluoglu-Cotul E., Arca A., Madak-Erdogan Z. (2019). Crosstalk between Estrogen Signaling and Breast Cancer Metabolism. Trends Endocrinol. Metab..

[22] Warburg O., Wind F., Negelein E. (1927). The Metabolism of Tumors in the Body. J. Gen. Physiol..

[23] Isidoro A., Casado E., Redondo A., Acebo P., Espinosa E., Alonso A.M., Cejas P., Hardisson D., Fresno Vara J.A., Belda-Iniesta C. (2005). Breast carcinomas fulfill the Warburg hypothesis and provide metabolic markers of cancer prognosis. Carcinogenesis.

[24] Fodor T., Szanto M., Abdul-Rahman O., Nagy L., Der A., Kiss B., Bai P. (2016). Combined Treatment of MCF-7 Cells with AICAR and Methotrexate, Arrests Cell Cycle and Reverses Warburg Metabolism through AMP-Activated Protein Kinase (AMPK) and FOXO1. PLoS ONE.

[25] Elia I., Schmieder R., Christen S., Fendt S.M. (2016). Organ-Specific Cancer Metabolism and Its Potential for Therapy. Handb. Exp. Pharmacol..

[26] Cha Y.J., Kim E.S., Koo J.S. (2018). Amino Acid Transporters and Glutamine Metabolism in Breast Cancer. Int. J. Mol. Sci..

[27] Poulose N., Mills I.G., Steele R.E. (2018). The impact of transcription on metabolism in prostate and breast cancers. Endocr. Relat. Cancer.

[28] Saavedra-Garcia P., Nichols K., Mahmud Z., Fan L.Y., Lam E.W. (2018). Unravelling the role of fatty acid metabolism in cancer through the FOXO3-FOXM1 axis. Mol. Cell Endocrinol..

[29] Blucher C., Stadler S.C. (2017). Obesity and Breast Cancer: Current Insights on the Role of Fatty Acids and Lipid Metabolism in Promoting Breast Cancer Growth and Progression. Front. Endocrinol. (Lausanne).

[30] Thakur S.B., Horvat J.V., Hancu I., Sutton O.M., Bernard-Davila B., Weber M., Oh J.H., Marino M.A., Avendano D., Leithner D. (2019). Quantitative in vivo proton MR spectroscopic assessment of lipid metabolism: Value for breast cancer diagnosis and prognosis. J. Magn. Reson. Imaging.

[31] Cha Y.J., Kim H.M., Koo J.S. (2017). Expression of Lipid Metabolism-Related Proteins Differs between Invasive Lobular Carcinoma and Invasive Ductal Carcinoma. Int. J. Mol. Sci..

[32] Possemato R., Marks K.M., Shaul Y.D., Pacold M.E., Kim D., Birsoy K., Sethumadhavan S., Woo H.K., Jang H.G., Jha A.K. (2011). Functional genomics reveal that the serine synthesis pathway is essential in breast cancer. Nature.

[33] van Geldermalsen M., Quek L.E., Turner N., Freidman N., Pang A., Guan Y.F., Krycer J.R., Ryan R., Wang Q., Holst J. (2018). Benzylserine inhibits breast cancer cell growth by disrupting intracellular amino acid homeostasis and triggering amino acid response pathways. BMC Cancer.

[34] Du T., Zhu L., Levine K.M., Tasdemir N., Lee A.V., Vignali D.A.A., Houten B.V., Tseng G.C., Oesterreich S. (2018). Invasive lobular and ductal breast carcinoma differ in immune response, protein translation efficiency and metabolism. Sci. Rep..

[35] Schito L., Rey S. (2017). Hypoxic pathobiology of breast cancer metastasis. Biochim. Biophys. Acta Rev. Cancer.

[36] Nie C., Lv H., Bie L., Hou H., Chen X. (2018). Hypoxia-inducible factor 1-alpha expression correlates with response to neoadjuvant chemotherapy in women with breast cancer. Medicine (Baltimore).

[37] Craze M.L., Cheung H., Jewa N., Coimbra N.D.M., Soria D., El-Ansari R., Aleskandarany M.A., Wai Cheng K., Diez-Rodriguez M., Nolan C.C. (2018). MYC regulation of glutamine-proline regulatory axis is key in luminal B breast cancer. Br. J. Cancer.

[38] Kim S., Kim D.H., Jung W.H., Koo J.S. (2013). Expression of glutamine metabolism-related proteins according to molecular subtype of breast cancer. Endocr. Relat. Cancer.

[39] El Ansari R., McIntyre A., Craze M.L., Ellis I.O., Rakha E.A., Green A.R. (2018). Altered glutamine metabolism in breast cancer; subtype dependencies and alternative adaptations. Histopathology.

[40] Maiese K. (2017). Moving to the Rhythm with Clock (Circadian) Genes, Autophagy, mTOR, and SIRT1 in Degenerative Disease and Cancer. Curr. Neurovasc. Res..

[41] Ranhotra H.S. (2018). The estrogen-related receptors in metabolism and cancer: Newer insights. J. Recept Signal. Transduct. Res..

[42] Raphael J., Desautels D., Pritchard K.I., Petkova E., Shah P.S. (2018). Phosphoinositide 3-kinase inhibitors in advanced breast cancer: A systematic review and meta-analysis. Eur. J. Cancer.

[43] Keegan N.M., Gleeson J.P., Hennessy B.T., Morris P.G. (2018). PI3K inhibition to overcome endocrine resistance in breast cancer. Expert Opin. Investig. Drugs.

[44] Hadad S.M., Baker L., Quinlan P.R., Robertson K.E., Bray S.E., Thomson G., Kellock D., Jordan L.B., Purdie C.A., Hardie D.G. (2009). Histological evaluation of AMPK signalling in primary breast cancer. BMC Cancer.

[45] Laderoute K.R., Calaoagan J.M., Chao W.R., Dinh D., Denko N., Duellman S., Kalra J., Liu X., Papandreou I., Sambucetti L. (2014). 5′-AMP-activated protein kinase (AMPK) supports the growth of aggressive experimental human breast cancer tumors. J. Biol. Chem..

[46] Sotgia F., Whitaker-Menezes D., Martinez-Outschoorn U.E., Salem A.F., Tsirigos A., Lamb R., Sneddon S., Hulit J., Howell A., Lisanti M.P. (2012). Mitochondria “fuel” breast cancer metabolism: Fifteen markers of mitochondrial biogenesis label epithelial cancer cells, but are excluded from adjacent stromal cells. Cell Cycle.

[47] Fuchinoue F., Hirotani Y., Nakanishi Y., Yamaguchi H., Nishimaki H., Noda H., Tang X.Y., Iizuka M., Amano S., Sugitani M. (2015). Overexpression of PGC1alpha and accumulation of p62 in apocrine carcinoma of the breast. Pathol. Int..

[48] Lim S., Liu H., Madeira da Silva L., Arora R., Liu Z., Phillips J.B., Schmitt D.C., Vu T., McClellan S., Lin Y. (2016). Immunoregulatory Protein B7-H3 Reprograms Glucose Metabolism in Cancer Cells by ROS-Mediated Stabilization of HIF1alpha. Cancer Res..

[49] Migneco G., Whitaker-Menezes D., Chiavarina B., Castello-Cros R., Pavlides S., Pestell R.G., Fatatis A., Flomenberg N., Tsirigos A., Howell A. (2010). Glycolytic cancer associated fibroblasts promote breast cancer tumor growth, without a measurable increase in angiogenesis: Evidence for stromal-epithelial metabolic coupling. Cell Cycle.

[50] Huang R., Zong X. (2017). Aberrant cancer metabolism in epithelial-mesenchymal transition and cancer metastasis: Mechanisms in cancer progression. Crit. Rev. Oncol. Hematol..

[51] Kondaveeti Y., Guttilla Reed I.K., White B.A. (2015). Epithelial-mesenchymal transition induces similar metabolic alterations in two independent breast cancer cell lines. Cancer Lett..

[52] Choi J., Cha Y.J., Koo J.S. (2018). Adipocyte biology in breast cancer: From silent bystander to active facilitator. Prog. Lipid Res..

[53] Hidayat K., Yang C.M., Shi B.M. (2018). Body fatness at a young age, body fatness gain and risk of breast cancer: Systematic review and meta-analysis of cohort studies. Obes. Rev..

[54] Martin S.D., McGee S.L. (2018). Metabolic reprogramming in type 2 diabetes and the development of breast cancer. J. Endocrinol..

[55] Liu L.N., Lin Y.C., Miaskowski C., Chen S.C., Chen M.L. (2017). Association between changes in body fat and disease progression after breast cancer surgery is moderated by menopausal status. BMC Cancer.

[56] Sun H., Zou J., Chen L., Zu X., Wen G., Zhong J. (2017). Triple-negative breast cancer and its association with obesity. Mol. Clin. Oncol..

[57] Avgerinos K.I., Spyrou N., Mantzoros C.S., Dalamaga M. (2018). Obesity and Cancer Risk: Emerging biological mechanisms and perspectives. Metabolism.

[58] Dennison J.B., Molina J.R., Mitra S., González-Angulo A.M., Balko J.M., Kuba M.G., Sanders M.E., Pinto J.A., Gómez H.L., Arteaga C.L. (2013). Lactate dehydrogenase B: A metabolic marker of response to neoadjuvant chemotherapy in breast cancer. Clin. Cancer Res..

[59] Zaal E.A., Berkers C.R. (2018). The Influence of Metabolism on Drug Response in Cancer. Front. Oncol..

[60] Palomeras S., Ruiz-Martinez S., Puig T. (2018). Targeting Breast Cancer Stem Cells to Overcome Treatment Resistance. Molecules.

[61] Sinn H.P., Kreipe H. (2013). A Brief Overview of the WHO Classification of Breast Tumors, 4th Edition, Focusing on Issues and Updates from the 3rd Edition. Breast Care (Basel).

[62] Warner M., Gustafsson J.A. (2014). On estrogen, cholesterol metabolism, and breast cancer. N. Engl. J. Med..

[63] Hultsch S., Kankainen M., Paavolainen L., Kovanen R.M., Ikonen E., Kangaspeska S., Pietiainen V., Kallioniemi O. (2018). Association of tamoxifen resistance and lipid reprogramming in breast cancer. BMC Cancer.

[64] Munir M.T., Ponce C., Powell C.A., Tarafdar K., Yanagita T., Choudhury M., Gollahon L.S., Rahman S.M. (2018). The contribution of cholesterol and epigenetic changes to the pathophysiology of breast cancer. J. Steroid Biochem. Mol. Biol..

[65] McDonnell D.P., Park S., Goulet M.T., Jasper J., Wardell S.E., Chang C.Y., Norris J.D., Guyton J.R., Nelson E.R. (2014). Obesity, cholesterol metabolism, and breast cancer pathogenesis. Cancer Res..

[66] Nelson E.R., Chang C.Y., McDonnell D.P. (2014). Cholesterol and breast cancer pathophysiology. Trends Endocrinol. Metab..

[67] Wu Q., Ishikawa T., Sirianni R., Tang H., McDonald J.G., Yuhanna I.S., Thompson B., Girard L., Mineo C., Brekken R.A. (2013). 27-Hydroxycholesterol promotes cell-autonomous, ER-positive breast cancer growth. Cell Rep..

[68] Mokarram P., Alizadeh J., Razban V., Barazeh M., Solomon C., Kavousipour S. (2017). Interconnection of Estrogen/Testosterone Metabolism and Mevalonate Pathway in Breast and Prostate Cancers. Curr. Mol. Pharmacol..

[69] Chen W., Zhu L., Yu X., Fu Q., Xu W., Wang P. (2018). Quantitative assessment of metabolic tumor burden in molecular subtypes of primary breast cancer with FDG PET/CT. Diagn. Interv. Radiol..

[70] Lee S.J., Chung M.S., Shin S.J., Choi Y.Y. (2018). Correlation of tumor uptake on breast-specific gamma imaging and fluorodeoxyglucose PET/CT with molecular subtypes of breast cancer. Medicine (Baltimore).

[71] Incoronato M., Grimaldi A.M., Cavaliere C., Inglese M., Mirabelli P., Monti S., Ferbo U., Nicolai E., Soricelli A., Catalano O.A. (2018). Relationship between functional imaging and immunohistochemical markers and prediction of breast cancer subtype: A PET/MRI study. Eur. J. Nucl. Med. Mol. Imaging.

[72] Mullarky E., Lairson L.L., Cantley L.C., Lyssiotis C.A. (2016). A novel small-molecule inhibitor of 3-phosphoglycerate dehydrogenase. Mol. Cell Oncol..

[73] Choi J., Kim E.S., Koo J.S. (2018). Expression of Pentose Phosphate Pathway-Related Proteins in Breast Cancer. Dis. Markers.

[74] Kim S.K., Jung W.H., Koo J.S. (2014). Differential expression of enzymes associated with serine/glycine metabolism in different breast cancer subtypes. PLoS ONE.

[75] Kalhan S.C., Hanson R.W. (2012). Resurgence of serine: An often neglected but indispensable amino Acid. J. Biol. Chem..

[76] Bostner J., Alayev A., Berman A.Y., Fornander T., Nordenskjold B., Holz M.K., Stal O. (2018). Raptor localization predicts prognosis and tamoxifen response in estrogen receptor-positive breast cancer. Breast Cancer Res. Treat..

[77] Shea M.P., O’Leary K.A., Wegner K.A., Vezina C.M., Schuler L.A. (2018). High collagen density augments mTOR-dependent cancer stem cells in ERalpha+ mammary carcinomas, and increases mTOR-independent lung metastases. Cancer Lett..

[78] Schettini F., Buono G., Trivedi M.V., De Placido S., Arpino G., Giuliano M. (2017). PI3K/mTOR Inhibitors in the Treatment of Luminal Breast Cancer. Why, When and to Whom?. Breast Care (Basel).

[79] Pavlides S., Whitaker-Menezes D., Castello-Cros R., Flomenberg N., Witkiewicz A.K., Frank P.G., Casimiro M.C., Wang C., Fortina P., Addya S. (2009). The reverse Warburg effect: Aerobic glycolysis in cancer associated fibroblasts and the tumor stroma. Cell Cycle.

[80] Bonuccelli G., Whitaker-Menezes D., Castello-Cros R., Pavlides S., Pestell R.G., Fatatis A., Witkiewicz A.K., Vander Heiden M.G., Migneco G., Chiavarina B. (2010). The reverse Warburg effect: Glycolysis inhibitors prevent the tumor promoting effects of caveolin-1 deficient cancer associated fibroblasts. Cell Cycle.

[81] De Luca A., Fiorillo M., Peiris-Pagès M., Ozsvari B., Smith D.L., Sanchez-Alvarez R., Martinez-Outschoorn U.E., Cappello A.R., Pezzi V., Lisanti M.P. (2015). Mitochondrial biogenesis is required for the anchorage-independent survival and propagation of stem-like cancer cells. Oncotarget.

[82] Choi J., Kim D.H., Jung W.H., Koo J.S. (2013). Metabolic interaction between cancer cells and stromal cells according to breast cancer molecular subtype. Breast Cancer Res..

[83] Kim S., Kim D.H., Jung W.H., Koo J.S. (2013). Metabolic phenotypes in triple-negative breast cancer. Tumour Biol..

[84] Feng W., Gentles A., Nair R.V., Huang M., Lin Y., Lee C.Y., Cai S., Scheeren F.A., Kuo A.H., Diehn M. (2014). Targeting unique metabolic properties of breast tumor initiating cells. Stem Cells.

[85] Fiorillo M., Peiris-Pagès M., Sanchez-Alvarez R., Bartella L., Di Donna L., Dolce V., Sindona G., Sotgia F., Cappello A.R., Lisanti M.P. (2018). Bergamot natural products eradicate cancer stem cells (CSCs) by targeting mevalonate, Rho-GDI-signalling and mitochondrial metabolism. Biochim. Biophys. Acta.

[86] Ozsvari B., Fiorillo M., Bonuccelli G., Cappello A.R., Frattaruolo L., Sotgia F., Trowbridge R., Foster R., Lisanti M.P. (2017). Mitoriboscins: Mitochondrial-based therapeutics targeting cancer stem cells (CSCs), bacteria and pathogenic yeast. Oncotarget.

[87] Fiorillo M., Lamb R., Tanowitz H.B., Cappello A.R., Martinez-Outschoorn U.E., Sotgia F., Lisanti M.P. (2016). Bedaquiline, an FDA-approved antibiotic, inhibits mitochondrial function and potently blocks the proliferative expansion of stem-like cancer cells (CSCs). Aging (Albany NY).

[88] Zhou Y., Xu Z., Quan D., Zhang F., Zhang H., Xiao T., Hou S., Qiao H., Harismendy O., Wang J.Y.J. (2018). Nuclear respiratory factor 1 promotes spheroid survival and mesenchymal transition in mammary epithelial cells. Oncogene.

[89] Rabinovich I., Sebastiao A.P.M., Lima R.S., Urban C.A., Junior E.S., Anselmi K.F., Elifio-Esposito S., De Noronha L., Moreno-Amaral A.N. (2018). Cancer stem cell markers ALDH1 and CD44+/CD24− phenotype and their prognosis impact in invasive ductal carcinoma. Eur J. Histochem..

[90] Rycaj K., Tang D.G. (2015). Cell-of-Origin of Cancer versus Cancer Stem Cells: Assays and Interpretations. Cancer Res..

[91] Kovács T., Mikó E., Vida A., Sebő É., Toth J., Csonka T., Boratkó A., Ujlaki G., Lente G., Kovács P. (2019). Cadaverine, a metabolite of the microbiome, reduces breast cancer aggressiveness through trace amino acid receptors. Sci. Rep..

[92] Ozsvari B., Sotgia F., Lisanti M.P. (2017). A new mutation-independent approach to cancer therapy: Inhibiting oncogenic RAS and MYC, by targeting mitochondrial biogenesis. Aging (Albany NY).

[93] Lamb R., Ozsvari B., Lisanti C.L., Tanowitz H.B., Howell A., Martinez-Outschoorn U.E., Sotgia F., Lisanti M.P. (2015). Antibiotics that target mitochondria effectively eradicate cancer stem cells, across multiple tumor types: Treating cancer like an infectious disease. Oncotarget.

[94] Lanning N.J., Castle J.P., Singh S.J., Leon A.N., Tovar E.A., Sanghera A., MacKeigan J.P., Filipp F.V., Graveel C.R. (2017). Metabolic profiling of triple-negative breast cancer cells reveals metabolic vulnerabilities. Cancer Metab..

[95] Winnike J.H., Stewart D.A., Pathmasiri W.W., McRitchie S.L., Sumner S.J. (2018). Stable Isotope-Resolved Metabolomic Differences between Hormone-Responsive and Triple-Negative Breast Cancer Cell Lines. Int. J. Breast Cancer.

[96] Morris B.A., Burkel B., Ponik S.M., Fan J., Condeelis J.S., Aguirre-Ghiso J.A., Castracane J., Denu J.M., Keely P.J. (2016). Collagen Matrix Density Drives the Metabolic Shift in Breast Cancer Cells. EBioMedicine.

[97] Gao D., Fish E.N. (2018). Chemokines in breast cancer: Regulating metabolism. Cytokine.

[98] Jones F.A. (2018). Physiology of the Gastrointestinal Tract.

[99] Chen J., Domingue J.C., Sears C.L. (2017). Microbiota dysbiosis in select human cancers: Evidence of association and causality. Semin. Immunol..

[100] Mima K., Nakagawa S., Sawayama H., Ishimoto T., Imai K., Iwatsuki M., Hashimoto D., Baba Y., Yamashita Y.I., Yoshida N. (2017). The microbiome and hepatobiliary-pancreatic cancers. Cancer Lett..

[101] Mao Q., Jiang F., Yin R., Wang J., Xia W., Dong G., Ma W., Yang Y., Xu L., Hu J. (2018). Interplay between the lung microbiome and lung cancer. Cancer Lett..

[102] Bai J., Behera M., Bruner D.W. (2018). The gut microbiome, symptoms, and targeted interventions in children with cancer: A systematic review. Support. Care Cancer.

[103] Biragyn A., Ferrucci L. (2018). Gut dysbiosis: A potential link between increased cancer risk in ageing and inflammaging. Lancet Oncol..

[104] Potgens S.A., Brossel H., Sboarina M., Catry E., Cani P.D., Neyrinck A.M., Delzenne N.M., Bindels L.B. (2018). Klebsiella oxytoca expands in cancer cachexia and acts as a gut pathobiont contributing to intestinal dysfunction. Sci. Rep..

[105] Xuan C., Shamonki J.M., Chung A., Dinome M.L., Chung M., Sieling P.A., Lee D.J. (2014). Microbial dysbiosis is associated with human breast cancer. PLoS ONE.

[106] Urbaniak C., Gloor G.B., Brackstone M., Scott L., Tangney M., Reid G. (2016). The Microbiota of Breast Tissue and Its Association with Breast Cancer. Appl. Environ. Microbiol..

[107] Banerjee S., Wei Z., Tan F., Peck K.N., Shih N., Feldman M., Rebbeck T.R., Alwine J.C., Robertson E.S. (2015). Distinct microbiological signatures associated with triple negative breast cancer. Sci. Rep..

[108] Chan A.A., Bashir M., Rivas M.N., Duvall K., Sieling P.A., Pieber T.R., Vaishampayan P.A., Love S.M., Lee D.J. (2016). Characterization of the microbiome of nipple aspirate fluid of breast cancer survivors. Sci. Rep..

[109] Hieken T.J., Chen J., Hoskin T.L., Walther-Antonio M., Johnson S., Ramaker S., Xiao J., Radisky D.C., Knutson K.L., Kalar K.R. (2016). The Microbiome of Aseptically Collected Human Breast Tissue in Benign and Malignant Disease. Sci. Rep..

[110] Urbaniak C., Cummins J., Brackstone M., Macklaim J.M., Gloor G.B., Baban C.K., Scott L., O’Hanlon D.M., Burton J.P., Francis K.P. (2014). Microbiota of human breast tissue. Appl. Environ. Microbiol..

[111] Wang H., Altemus J., Niazi F., Green H., Calhoun B.C., Sturgis C., Grobmyer S.R., Eng C. (2017). Breast tissue, oral and urinary microbiomes in breast cancer. Oncotarget.

[112] hompson K.J., Ingle J.N., Tang X., Chia N., Jeraldo P.R., Walther-Antonio M.R., Kandimalla K.K., Johnson S., Yao J.Z., Harrington S.C. (2017). A comprehensive analysis of breast cancer microbiota and host gene expression. PLoS ONE.

[113] Banerjee S., Tian T., Wei Z., Shih N., Feldman M.D., Peck K.N., DeMichele A.M., Alwine J.C., Robertson E.S. (2018). Distinct Microbial Signatures Associated With Different Breast Cancer Types. Front. Microbiol..

[114] Costantini L., Magno S., Albanese D., Donati C., Molinari R., Filippone A., Masetti R., Merendino N. (2018). Characterization of human breast tissue microbiota from core needle biopsies through the analysis of multi hypervariable 16S-rRNA gene regions. Sci. Rep..

[115] Meng S., Chen B., Yang J., Wang J., Zhu D., Meng Q., Zhang L. (2018). Study of Microbiomes in Aseptically Collected Samples of Human Breast Tissue Using Needle Biopsy and the Potential Role of in situ Tissue Microbiomes for Promoting Malignancy. Front. Oncol..

[116] Flores R., Shi J., Fuhrman B., Xu X., Veenstra T.D., Gail M.H., Gajer P., Ravel J., Goedert J.J. (2012). Fecal microbial determinants of fecal and systemic estrogens and estrogen metabolites: A cross-sectional study. J. Transl Med..

[117] Fuhrman B.J., Feigelson H.S., Flores R., Gail M.H., Xu X., Ravel J., Goedert J.J. (2014). Associations of the fecal microbiome with urinary estrogens and estrogen metabolites in postmenopausal women. J. Clin. Endocrinol. Metab..

[118] Goedert J.J., Jones G., Hua X., Xu X., Yu G., Flores R., Falk R.T., Gail M.H., Shi J., Ravel J. (2015). Investigation of the association between the fecal microbiota and breast cancer in postmenopausal women: A population-based case-control pilot study. J. Natl, Cancer Inst..

[119] Goedert J.J., Hua X., Bielecka A., Okayasu I., Milne G.L., Jones G.S., Fujiwara M., Sinha R., Wan Y., Xu X. (2018). Postmenopausal breast cancer and oestrogen associations with the IgA-coated and IgA-noncoated faecal microbiota. Br. J. Cancer.

[120] Miko E., Vida A., Kovácsm T., Ujlakim G., Trencsényim G., Márton J., Sári Z., Kovács P., Boratkó A., Hujber Z. (2018). Lithocholic acid, a bacterial metabolite reduces breast cancer cell proliferation and aggressiveness. Biochim. Biophys. Acta.

[121] Luu T.H., Michel C., Bard J.M., Dravet F., Nazih H., Bobin-Dubigeon C. (2017). Intestinal Proportion of Blautia sp. is Associated with Clinical Stage and Histoprognostic Grade in Patients with Early-Stage Breast Cancer. Nutr. Cancer.

[122] Zhu J., Liao M., Yao Z., Liang W., Li Q., Liu J., Yang H., Ji Y., Wei W., Tan A. (2018). Breast cancer in postmenopausal women is associated with an altered gut metagenome. Microbiome.

[123] Robinson K.M., Crabtree J., Mattick J.S., Anderson K.E., Dunning Hotopp J.C. (2017). Distinguishing potential bacteria-tumor associations from contamination in a secondary data analysis of public cancer genome sequence data. Microbiome.

[124] Noguti J., Lee D.J. (2019). Association of microbes with breast cancer. Microbiome and Cancer.

[125] Fruge A.D., Van der Pol W., Rogers L.Q., Morrow C.D., Tsuruta Y., Demark-Wahnefried W. (2018). Fecal Akkermansia muciniphila Is Associated with Body Composition and Microbiota Diversity in Overweight and Obese Women with Breast Cancer Participating in a Presurgical Weight Loss Trial. J. Acad. Nutr. Diet..

[126] Velicer C.M., Heckbert S.R., Lampe J.W., Potter J.D., Robertson C.A., Taplin S.H. (2004). Antibiotic use in relation to the risk of breast cancer. JAMA.

[127] Velicer C.M., Heckbert S.R., Rutter C., Lampe J.W., Malone K. (2006). Association between antibiotic use prior to breast cancer diagnosis and breast tumour characteristics (United States). Cancer Causes Control.

[128] Friedman G.D., Oestreicher N., Chan J., Quesenberry C.P., Udaltsova N., Habel L.A. (2006). Antibiotics and risk of breast cancer: Up to 9 years of follow-up of 2.1 million women. Cancer Epidemiol. Biomarkers Prev..

[129] Wirtz H.S., Buist D.S.M., Gralow J.R., Barlow W.E., Gray S., Chubak J., Yu O., Bowles E.J.A., Fujii M., Boudreau D.M. (2013). Frequent antibiotic use and second breast cancer events. Cancer Epidemiol. Biomarkers Prev..

[130] Tamim H.M., Hanley J.A., Hajeer A.H., Boivin J.F., Collet J.P. (2008). Risk of breast cancer in relation to antibiotic use. Pharmacoepidemiol. Drug Saf..

[131] Satram-Hoang S., Moran E.M., Anton-Culver H., Burras R.W., Heimann T.M., Boggio I., Dykstra-Long G.R., Wood P.A., Zulka R., Hufnagel G. (2010). A pilot study of male breast cancer in the Veterans Affairs healthcare system. J. Environ. Pathol. Toxicol. Oncol..

[132] Kirkup B., McKee A., Makin K., Paveley J., Caim S., Alcon-Giner C., Leclaire C., Dalby M., Le Gall G., Andrusaite A. (2019). Perturbation of the gut microbiota by antibiotics results in accelerated breast tumour growth and metabolic dysregulation. BioRxiv.

[133] Miko E., Vida A., Bai P. (2016). Translational aspects of the microbiome-to be exploited. Cell Biol. Toxicol..

[134] Tao J., Li S., Gan R.Y., Zhao C.N., Meng X., Li H.B. (2019). Targeting gut microbiota with dietary components on cancer: Effects and potential mechanisms of action. Crit. Rev. Food Sci. Nutr..

[135] Di Ciaula A., Wang D.Q., Molina-Molina E., Lunardi Baccetto R., Calamita G., Palmieri V.O., Portincasa P. (2017). Bile Acids and Cancer: Direct and Environmental-Dependent Effects. Ann. Hepatol..

[136] Wikoff W.R., Anfora A.T., Liu J., Schultz P.G., Lesley S.A., Peters E.C., Siuzdak G. (2009). Metabolomics analysis reveals large effects of gut microflora on mammalian blood metabolites. Proc. Natl. Acad. Sci. USA.

[137] Dumas M.E. (2011). The microbial-mammalian metabolic axis: Beyond simple metabolism. Cell Metab..

[138] Burcelin R., Serino M., Chabo C., Garidou L., Pomie C., Courtney M., Amar J., Bouloumie A. (2013). Metagenome and metabolism: The tissue microbiota hypothesis. Diabetes Obes. Metab..

[139] Puertollano E., Kolida S., Yaqoob P. (2014). Biological significance of short-chain fatty acid metabolism by the intestinal microbiome. Curr. Opin. Clin. Nutr. Metab. Care.

[140] Goldberg A.A., Beach A., Davies G.F., Harkness T.A., Leblanc A., Titorenko V.I. (2011). Lithocholic bile acid selectively kills neuroblastoma cells, while sparing normal neuronal cells. Oncotarget.

[141] Goldberg A.A., Titorenko V.I., Beach A., Sanderson J.T. (2013). Bile acids induce apoptosis selectively in androgen-dependent and -independent prostate cancer cells. PeerJ.

[142] Gafar A.A., Draz H.M., Goldberg A.A., Bashandy M.A., Bakry S., Khalifa M.A., AbuShair W., Titorenko V.I., Sanderson J.T. (2016). Lithocholic acid induces endoplasmic reticulum stress, autophagy and mitochondrial dysfunction in human prostate cancer cells. PeerJ.

[143] Luu T.H., Bard J.M., Carbonnelle D., Chaillou C., Huvelin J.M., Bobin-Dubigeon C., Nazih H. (2018). Lithocholic bile acid inhibits lipogenesis and induces apoptosis in breast cancer cells. Cell Oncol. (Dordr).

[144] Thirunavukkarasan M., Wang C., Rao A., Hind T., Teo Y.R., Siddiquee A.A., Goghari M.A.I., Kumar A.P., Herr D.R. (2017). Short-chain fatty acid receptors inhibit invasive phenotypes in breast cancer cells. PLoS ONE.

[145] Dabek M., McCrae S.I., Stevens V.J., Duncan S.H., Louis P. (2008). Distribution of beta-glucosidase and beta-glucuronidase activity and of beta-glucuronidase gene gus in human colonic bacteria. FEMS Microbiol. Ecol..

[146] McIntosh F.M., Maison N., Holtrop G., Young P., Stevens V.J., Ince J., Johnstone A.M., Lobley G.E., Flint H.J., Louis P. (2012). Phylogenetic distribution of genes encoding beta-glucuronidase activity in human colonic bacteria and the impact of diet on faecal glycosidase activities. Environ. Microbiol..

[147] Gloux K., Berteau O., El Oumami H., Beguet F., Leclerc M., Dore J. (2011). A metagenomic beta-glucuronidase uncovers a core adaptive function of the human intestinal microbiome. Proc. Natl. Acad. Sci. USA.

[148] Radde B.N., Ivanova M.M., Mai H.X., Salabei J.K., Hill B.G., Klinge C.M. (2015). Bioenergetic differences between MCF-7 and T47D breast cancer cells and their regulation by oestradiol and tamoxifen. Biochem. J..

[149] Radde B.N., Ivanova M.M., Mai H.X., Alizadeh-Rad N., Piell K., Van Hoose P., Cole M.P., Muluhngwi P., Kalbfleisch T.S., Rouchka E.C. (2016). Nuclear respiratory factor-1 and bioenergetics in tamoxifen-resistant breast cancer cells. Exp. Cell Res..

[150] Sotgia F., Lisanti M.P. (2017). Mitochondrial mRNA transcripts predict overall survival, tumor recurrence and progression in serous ovarian cancer: Companion diagnostics for cancer therapy. Oncotarget.

[151] Zacksenhaus E., Shrestha M., Liu J.C., Vorobieva I., Chung P.E.D., Ju Y., Nir U., Jiang Z. (2017). Mitochondrial OXPHOS Induced by RB1 Deficiency in Breast Cancer: Implications for Anabolic Metabolism, Stemness, and Metastasis. Trends Cancer.

[152] Maximov P.Y., Abderrahman B., Curpan R.F., Hawsawi Y.M., Fan P., Jordan V.C. (2018). A unifying biology of sex steroid-induced apoptosis in prostate and breast cancers. Endocr. Relat. Cancer.

[153] Al-Howail H.A., Hakami H.A., Al-Otaibi B., Al-Mazrou A., Daghestani M.H., Al-Jammaz I., Al-Khalaf H.H., Aboussekhra A. (2016). PAC down-regulates estrogen receptor alpha and suppresses epithelial-to-mesenchymal transition in breast cancer cells. BMC Cancer.

[154] Bouris P., Skandalis S.S., Piperigkou Z., Afratis N., Karamanou K., Aletras A.J., Moustakas A., Theocharis A.D., Karamanos N.K. (2015). Estrogen receptor alpha mediates epithelial to mesenchymal transition, expression of specific matrix effectors and functional properties of breast cancer cells. Matrix Biol..

[155] Derrien M., Vaughan E.E., Plugge C.M., de Vos W.M. (2004). Akkermansia muciniphila gen. nov., sp. nov., a human intestinal mucin-degrading bacterium. Int. J. Syst. Evol. Microbiol..

[156] Louis P., Young P., Holtrop G., Flint H.J. (2010). Diversity of human colonic butyrate-producing bacteria revealed by analysis of the butyryl-CoA:acetate CoA-transferase gene. Environ. Microbiol..

[157] Reichardt N., Duncan S.H., Young P., Belenguer A., McWilliam Leitch C., Scott K.P., Flint H.J., Louis P. (2014). Phylogenetic distribution of three pathways for propionate production within the human gut microbiota. ISME J..

[158] Rodrigues M.F., Carvalho E., Pezzuto P., Rumjanek F.D., Amoedo N.D. (2015). Reciprocal modulation of histone deacetylase inhibitors sodium butyrate and trichostatin A on the energy metabolism of breast cancer cells. J. Cell Biochem..

[159] Salimi V., Shahsavari Z., Safizadeh B., Hosseini A., Khademian N., Tavakoli-Yaraki M. (2017). Sodium butyrate promotes apoptosis in breast cancer cells through reactive oxygen species (ROS) formation and mitochondrial impairment. Lipids Health Dis..

[160] Arpaia N., Campbell C., Fan X., Dikiy S., van der Veeken J., deRoos P., Liu H., Cross J.R., Pfeffer K., Coffer P.J. (2013). Metabolites produced by commensal bacteria promote peripheral regulatory T-cell generation. Nature.

[161] Tan J., McKenzie C., Potamitis M., Thorburn A.N., Mackay C.R., Macia L. (2014). The role of short-chain Fatty acids in health and disease. Adv. Immunol..

[162] Yu X., Shahir A.M., Sha J., Feng Z., Eapen B., Nithianantham S., Das B., Karn J., Weinberg A., Bissada N.F. (2014). Short Chain Fatty Acids From Periodontal Pathogens Suppress HDACs, EZH2, and SUV39H1 to Promote Kaposi’s Sarcoma-Associated Herpesvirus Replication. J. Virol..

[163] Schulthess J., Pandey S., Capitani M., Rue-Albrecht K.C., Arnold I., Franchini F., Chomka A., Ilott N.E., Johnston D.G.W., Pires E. (2019). The Short Chain Fatty Acid Butyrate Imprints an Antimicrobial Program in Macrophages. Immunity.

[164] Ridlon J.M., Kang D.J., Hylemon P.B. (2006). Bile salt biotransformations by human intestinal bacteria. J. Lipid Res..

[165] Ridlon J.M., Harris S.C., Bhowmik S., Kang D.J., Hylemon P.B. (2016). Consequences of bile salt biotransformations by intestinal bacteria. Gut Microbes.

[166] Swales K.E., Korbonits M., Carpenter R., Walsh D.T., Warner T.D., Bishop-Bailey D. (2006). The farnesoid X receptor is expressed in breast cancer and regulates apoptosis and aromatase expression. Cancer Res..

[167] Tang X., Lin C.C., Spasojevic I., Iversen E.S., Chi J.T., Marks J.R. (2014). A joint analysis of metabolomics and genetics of breast cancer. Breast Cancer Res..

[168] Baker J.M., Al-Nakkash L., Herbst-Kralovetz M.M. (2017). Estrogen-gut microbiome axis: Physiological and clinical implications. Maturitas.

[169] Kwa M., Plottel C.S., Blaser M.J., Adams S. (2016). The Intestinal Microbiome and Estrogen Receptor-Positive Female Breast Cancer. J. Natl. Cancer Inst..

[170] Chen J.Q., Yager J.D. (2004). Estrogen’s effects on mitochondrial gene expression: Mechanisms and potential contributions to estrogen carcinogenesis. Ann. N. Y. Acad. Sci..

[171] Chen J.Q., Delannoy M., Cooke C., Yager J.D. (2004). Mitochondrial localization of ERalpha and ERbeta in human MCF7 cells. Am. J. Physiol. Endocrinol. Metab..

[172] Chen J.Q., Russo P.A., Cooke C., Russo I.H., Russo J. (2007). ERbeta shifts from mitochondria to nucleus during estrogen-induced neoplastic transformation of human breast epithelial cells and is involved in estrogen-induced synthesis of mitochondrial respiratory chain proteins. Biochim. Biophys. Acta.

[173] Sansone P., Savini C., Kurelac I., Chang Q., Amato L.B., Strillacci A., Stepanova A., Iommarini L., Mastroleo C., Daly L. (2017). Packaging and transfer of mitochondrial DNA via exosomes regulate escape from dormancy in hormonal therapy-resistant breast cancer. Proc. Natl. Acad. Sci. USA.

[174] Sastre-Serra J., Valle A., Company M.M., Garau I., Oliver J., Roca P. (2010). Estrogen down-regulates uncoupling proteins and increases oxidative stress in breast cancer. Free Radic. Biol. Med..

[175] Sastre-Serra J., Nadal-Serrano M., Pons D.G., Valle A., Garau I., Garcia-Bonafe M., Oliver J., Roca P. (2013). The oxidative stress in breast tumors of postmenopausal women is ERalpha/ERbeta ratio dependent. Free Radic. Biol. Med..

[176] Morrison D.J., Preston T. (2016). Formation of short chain fatty acids by the gut microbiota and their impact on human metabolism. Gut Microbes.

[177] Clausen M.R., Mortensen P.B., Bendtsen F. (1991). Serum levels of short-chain fatty acids in cirrhosis and hepatic coma. Hepatology.

[178] Jakobsdottir G., Bjerregaard J.H., Skovbjerg H., Nyman M. (2013). Fasting serum concentration of short-chain fatty acids in subjects with microscopic colitis and celiac disease: No difference compared with controls, but between genders. Scand. J. Gastroenterol..

[179] Ktsoyan Z.A., Mkrtchyan M.S., Zakharyan M.K., Mnatsakanyan A.A., Arakelova K.A., Gevorgyan Z.U., Sedrakyan A.M., Hovhannisyan A.I., Arakelyan A.A., Aminov R.I. (2016). Systemic Concentrations of Short Chain Fatty Acids Are Elevated in Salmonellosis and Exacerbation of Familial Mediterranean Fever. Front. Microbiol..

[180] Hopkins M.M., Meier K.E. (2017). Free Fatty Acid Receptors and Cancer: From Nutrition to Pharmacology. Handb. Exp. Pharmacol..

[181] Yonezawa T., Kobayashi Y., Obara Y. (2007). Short-chain fatty acids induce acute phosphorylation of the p38 mitogen-activated protein kinase/heat shock protein 27 pathway via GPR43 in the MCF-7 human breast cancer cell line. Cell Signal..

[182] Bindels L.B., Porporato P., Dewulf E.M., Verrax J., Neyrinck A.M., Martin J.C., Scott K.P., Buc Calderon P., Feron O., Muccioli G.G. (2012). Gut microbiota-derived propionate reduces cancer cell proliferation in the liver. Br. J. Cancer.

[183] Ivan J., Major E., Sipos A., Kovacs K., Horvath D., Tamas I., Bay P., Dombradi V., Lontay B. (2017). The Short-Chain Fatty Acid Propionate Inhibits Adipogenic Differentiation of Human Chorion-Derived Mesenchymal Stem Cells Through the Free Fatty Acid Receptor 2. Stem Cells Dev..

[184] Huang C.K., Chang P.H., Kuo W.H., Chen C.L., Jeng Y.M., Chang K.J., Shew J.Y., Hu C.M., Lee W.H. (2017). Adipocytes promote malignant growth of breast tumours with monocarboxylate transporter 2 expression via beta-hydroxybutyrate. Nat. Commun..

[185] Martinez-Outschoorn U.E., Lisanti M.P., Sotgia F. (2014). Catabolic cancer-associated fibroblasts transfer energy and biomass to anabolic cancer cells, fueling tumor growth. Semin. Cancer Biol..

[186] Long S.L., Gahan C.G.M., Joyce S.A. (2017). Interactions between gut bacteria and bile in health and disease. Mol. Aspects Med..

[187] Javitt N.B., Budai K., Miller D.G., Cahan A.C., Raju U., Levitz M. (1994). Breast-gut connection: Origin of chenodeoxycholic acid in breast cyst fluid. Lancet.

[188] Raju U., Levitz M., Javitt N.B. (1990). Bile acids in human breast cyst fluid: The identification of lithocholic acid. J. Clin. Endocrinol. Metab..

[189] Watanabe M., Houten S.M., Mataki C., Christoffolete M.A., Kim B.W., Sato H., Messaddeq N., Harney J.W., Ezaki O., Kodama T. (2006). Bile acids induce energy expenditure by promoting intracellular thyroid hormone activation. Nature.

[190] Thomas C., Gioiello A., Noriega L., Strehle A., Oury J., Rizzo G., Macchiarulo A., Yamamoto H., Mataki C., Pruzanski M. (2009). TGR5-mediated bile acid sensing controls glucose homeostasis. Cell Metab..

[191] Lefebvre P., Cariou B., Lien F., Kuipers F., Staels B. (2009). Role of bile acids and bile acid receptors in metabolic regulation. Physiol. Rev..

[192] Martinot E., Sedes L., Baptissart M., Lobaccaro J.M., Caira F., Beaudoin C., Volle D.H. (2017). Bile acids and their receptors. Mol. Aspects Med..

[193] Miller-Fleming L., Olin-Sandoval V., Campbell K., Ralser M. (2015). Remaining Mysteries of Molecular Biology: The Role of Polyamines in the Cell. J. Mol. Biol..

[194] Seiler N. (2004). Catabolism of polyamines. Amino Acids.

[195] de las Rivas B., Marcobal A., Carrascosa A.V., Munoz R. (2006). PCR detection of foodborne bacteria producing the biogenic amines histamine, tyramine, putrescine, and cadaverine. J. Food Prot..

[196] Loser C., Folsch U.R., Paprotny C., Creutzfeldt W. (1990). Polyamine concentrations in pancreatic tissue, serum, and urine of patients with pancreatic cancer. Pancreas.

[197] Loser C., Folsch U.R., Paprotny C., Creutzfeldt W. (1990). Polyamines in colorectal cancer. Evaluation of polyamine concentrations in the colon tissue, serum, and urine of 50 patients with colorectal cancer. Cancer..

[198] Vattai A., Akyol E., Kuhn C., Hofmann S., Heidegger H., von Koch F., Hermelink K., Wuerstlein R., Harbeck N., Mayr D. (2017). Increased trace amine-associated receptor 1 (TAAR1) expression is associated with a positive survival rate in patients with breast cancer. J. Cancer Res. Clin. Oncol..

[199] Bashiardes S., Tuganbaev T., Federici S., Elinav E. (2017). The microbiome in anti-cancer therapy. Semin. Immunol..

[200] Roy S., Trinchieri G. (2017). Microbiota: A key orchestrator of cancer therapy. Nat. Rev. Cancer.

[201] Manepalli S., Gandhi J.A., Ekhar V.V., Asplund M.B., Coelho C., Martinez L.R. (2013). Characterization of a cyclophosphamide-induced murine model of immunosuppression to study Acinetobacter baumannii pathogenesis. J. Med. Microbiol..

[202] Viaud S., Saccheri F., Mignot G., Yamazaki T., Daillère R., Hannani D., Enot D.P., Pfirschke C., Engblom C., Pittet M.J. (2013). The intestinal microbiota modulates the anticancer immune effects of cyclophosphamide. Science.

[203] Alexander J.L., Wilson I.D., Teare J., Marchesi J.R., Nicholson J.K., Kinross J.M. (2017). Gut microbiota modulation of chemotherapy efficacy and toxicity. Nat. Rev. Gastroenterol. Hepatol..

[204] Cox G., Koteva K., Wright G.D. (2014). An unusual class of anthracyclines potentiate Gram-positive antibiotics in intrinsically resistant Gram-negative bacteria. J. Antimicrob. Chemother..

[205] McCarron A.J., Armstrong C., Glynn G., Millar B.C., Rooney P.J., Goldsmith C.E., Xu J., Moore J.E. (2012). Antibacterial effects on acinetobacter species of commonly employed antineoplastic agents used in the treatment of haematological malignancies: An in vitro laboratory evaluation. Br. J. Biomed. Sci..

[206] Parajuli P., Pandey R.P., Nguyen T.H.T., Dhakal D., Sohng J.K. (2018). Substrate Scope of O-Methyltransferase from Streptomyces peucetius for Biosynthesis of Diverse Natural Products Methoxides. Appl. Biochem. Biotechnol..

[207] Dhakal D., Lim S.K., Kim D.H., Kim B.G., Yamaguchi T., Sohng J.K. (2018). Complete genome sequence of Streptomyces peucetius ATCC 27952, the producer of anticancer anthracyclines and diverse secondary metabolites. J. Biotechnol..

[208] Zabala D., Brana A.F., Florez A.B., Salas J.A., Mendez C. (2013). Engineering precursor metabolite pools for increasing production of antitumor mithramycins in Streptomyces argillaceus. Metab. Eng..

[209] Westman E.L., Canova M.J., Radhi I.J., Koteva K., Kireeva I., Waglechner N., Wright G.D. (2012). Bacterial inactivation of the anticancer drug doxorubicin. Chem. Biol..

[210] Bolourian A., Mojtahedi Z. (2018). Streptomyces, shared microbiome member of soil and gut, as ’old friends’ against colon cancer. FEMS Microbiol. Ecol..

[211] Yang J., Liu K.X., Qu J.M., Wang X.D. (2013). The changes induced by cyclophosphamide in intestinal barrier and microflora in mice. Eur. J. Pharmacol..

[212] Liu T., Wu Y., Wang L., Pang X., Zhao L., Yuan H., Zhang C. (2019). A More Robust Gut Microbiota in Calorie-Restricted Mice Is Associated with Attenuated Intestinal Injury Caused by the Chemotherapy Drug Cyclophosphamide. MBio.

[213] Xie J.H., Fan S.T., Nie S.P., Yu Q., Xiong T., Gong D., Xie M.Y. (2016). Lactobacillus plantarum NCU116 attenuates cyclophosphamide-induced intestinal mucosal injury, metabolism and intestinal microbiota disorders in mice. Food Funct..

[214] Daillere R., Vétizou M., Waldschmitt N., Yamazaki T., Isnard C., Poirier-Colame V., Duong C.P.M., Flament C., Lepage P., Roberti M.P. (2016). Enterococcus hirae and Barnesiella intestinihominis Facilitate Cyclophosphamide-Induced Therapeutic Immunomodulatory Effects. Immunity.

[215] Salva S., Marranzino G., Villena J., Aguero G., Alvarez S. (2014). Probiotic Lactobacillus strains protect against myelosuppression and immunosuppression in cyclophosphamide-treated mice. Int. Immunopharmacol..

[216] Hussein M.H., Schneider E.K., Elliott A.G., Han M., Reyes-Ortega F., Morris F., Blastovich M.A.T., Jasim R., Currie B., Mayo M. (2017). From Breast Cancer to Antimicrobial: Combating Extremely Resistant Gram-Negative “Superbugs” Using Novel Combinations of Polymyxin B with Selective Estrogen Receptor Modulators. Microb. Drug Resist..

[217] Scott S.A., Spencer C.T., O’Reilly M.C., Brown K.A., Lavieri R.R., Cho C.H., Jung D.I., Larock R.C., Brown H.A., Lindsley C.W. (2015). Discovery of desketoraloxifene analogues as inhibitors of mammalian, Pseudomonas aeruginosa, and NAPE phospholipase D enzymes. ACS Chem. Biol..

[218] Ho Sui S.J., Lo R., Fernandes A.R., Caulfield M.D., Lerman J.A., Xie L., Bourne P.E., Baillie D.L., Brinkman F.S. (2012). Raloxifene attenuates Pseudomonas aeruginosa pyocyanin production and virulence. Int. J. Antimicrob. Agents.

[219] Gerits E., Defraine V., Vandamme K., De Cremer K., De Brucker K., Thevissen K., Cammue B.P.A., Beullens S., Fauvart M., Verstraeten N. (2017). Repurposing Toremifene for Treatment of Oral Bacterial Infections. Antimicrob. Agents Chemother..

[220] Jacobs A.C., Didone L., Jobson J., Sofia M.K., Krysan D., Dunman P.M. (2013). Adenylate kinase release as a high-throughput-screening-compatible reporter of bacterial lysis for identification of antibacterial agents. Antimicrob. Agents Chemother..

[221] Luxo C., Jurado A.S., Custodio J.B., Madeira V.M. (2001). Toxic effects of tamoxifen on the growth and respiratory activity of Bacillus stearothermophilus. Toxicol. In Vitro.

[222] Poirot M., Silvente-Poirot S., Weichselbaum R.R. (2012). Cholesterol metabolism and resistance to tamoxifen. Curr. Opin. Pharmacol..

[223] Dou T.Y., Luan H.W., Liu X.B., Li S.Y., Du X.F., Yang L. (2015). Enzymatic hydrolysis of 7-xylosyltaxanes by an extracellular xylosidase from Cellulosimicrobium cellulans. Biotechnol. Lett..

[224] Zhou D.J., Pan J., Yu H.L., Zheng G.W., Xu J.H. (2013). Target-oriented discovery of a new esterase-producing strain Enterobacter sp. ECU1107 for whole cell-catalyzed production of (2S,3R)-3-phenylglycidate as a chiral synthon of Taxol. Appl. Microbiol. Biotechnol..

[225] Byrd C.A., Bornmann W., Erdjument-Bromage H., Tempst P., Pavletich N., Rosen N., Nathan C.F., Ding A. (1999). Heat shock protein 90 mediates macrophage activation by Taxol and bacterial lipopolysaccharide. Proc. Natl. Acad. Sci. USA.

[226] Oelschlaeger T.A., Tall B.D. (1997). Invasion of cultured human epithelial cells by Klebsiella pneumoniae isolated from the urinary tract. Infect. Immun..

[227] Garcia-Gonzalez A.P., Ritter A.D., Shrestha S., Andersen E.C., Yilmaz L.S., Walhout A.J.M. (2017). Bacterial Metabolism Affects the C. elegans Response to Cancer Chemotherapeutics. Cell.

[228] Scott T.A., Quintaneiro L.M., Norvaisas P., Lui P.P., Wilson M.P., Leung K.Y., Herrera-Dominguez L., Sudiwala S., Pessia A., Clayton P.T. (2017). Host-Microbe Co-metabolism Dictates Cancer Drug Efficacy in C. elegans. Cell.

[229] Geller L.T., Barzily-Rokni M., Danino T., Jonas O.H., Shental N., Nejman D., Gavert N., Zwang Y., Cooper Z.A., Shee K. (2017). Potential role of intratumor bacteria in mediating tumor resistance to the chemotherapeutic drug gemcitabine. Science.

[230] Vande Voorde J., Sabuncuoglu S., Noppen S., Hofer A., Ranjbarian F., Fieuws S., Balzarini J., Liekens S. (2014). Nucleoside-catabolizing enzymes in mycoplasma-infected tumor cell cultures compromise the cytostatic activity of the anticancer drug gemcitabine. J. Biol Chem..

[231] Lehouritis P., Cummins J., Stanton M., Murphy C.T., McCarthy F.O., Reid G., Urbaniak C., Byrne W.L., Tangney M. (2015). Local bacteria affect the efficacy of chemotherapeutic drugs. Sci. Rep..

[232] Geller L.T., Straussman R. (2018). Intratumoral bacteria may elicit chemoresistance by metabolizing anticancer agents. Mol. Cell Oncol..

[233] Sandrini M.P., Shannon O., Clausen A.R., Bjorck L., Piskur J. (2007). Deoxyribonucleoside kinases activate nucleoside antibiotics in severely pathogenic bacteria. Antimicrob. Agents Chemother..

[234] Sandrini M.P., Clausen A.R., On S.L., Aarestrup F.M., Munch-Petersen B., Piskur J. (2007). Nucleoside analogues are activated by bacterial deoxyribonucleoside kinases in a species-specific manner. J. Antimicrob. Chemother..

[235] Yuan L., Zhang S., Li H., Yang F., Mushtaq N., Ullah S., Shi Y., An C., Xu J. (2018). The influence of gut microbiota dysbiosis to the efficacy of 5-Fluorouracil treatment on colorectal cancer. Biomed. Pharmacother..

[236] Vanlancker E., Vanhoecke B., Smet R., Props R., Van de Wiele T. (2016). 5-Fluorouracil sensitivity varies among oral micro-organisms. J. Med. Microbiol..

[237] Singh V., Brecik M., Mukherjee R., Evans J.C., Svetlíková Z., Blaško J., Surade S., Blackburn J., Warner D.F., Mikušová K. (2015). The complex mechanism of antimycobacterial action of 5-fluorouracil. Chem. Biol..

[238] Hamouda N., Sano T., Oikawa Y., Ozaki T., Shimakawa M., Matsumoto K., Amagase K., Higuchi K., Kato S. (2017). Apoptosis, Dysbiosis and Expression of Inflammatory Cytokines are Sequential Events in the Development of 5-Fluorouracil-Induced Intestinal Mucositis in Mice. Basic Clin. Pharmacol. Toxicol..

[239] Li H.L., Lu L., Wang X.S., Qin L.Y., Wang P., Qiu S.P., Wu H., Huang F., Zhang B.B., Shi H.L. (2017). Alteration of Gut Microbiota and Inflammatory Cytokine/Chemokine Profiles in 5-Fluorouracil Induced Intestinal Mucositis. Front. Cell Infect. Microbiol..

[240] Yeung C.Y., Chan W.T., Jiang C.B., Cheng M.L., Liu C.Y., Chang S.W., Chiang Chiau J.S., Lee H.C. (2015). Amelioration of Chemotherapy-Induced Intestinal Mucositis by Orally Administered Probiotics in a Mouse Model. PLoS ONE.

[241] Kucuk C., Ozkan M., Akgun H., Muhtaroglu S., Sozuer E. (2005). The effect of granulocyte macrophage-colony stimulating factor on bacterial translocation after administration of 5-fluorouracil in rats. J. Surg. Res..

[242] Vida A., Kardos G., Kovacs T., Bodrogi B.L., Bai P. (2018). Deletion of poly(ADPribose) polymerase-1 changes the composition of the microbiome in the gut. Mol. Med. Rep..

[243] Larmonier C.B., Shehab K.W., Laubitz D., Jamwal D.R., Ghishan F.K., Kiela P.R. (2016). Transcriptional Reprogramming and Resistance to Colonic Mucosal Injury in Poly(ADP-ribose) Polymerase 1 (PARP1)-deficient Mice. J. Biol Chem..

[244] Zhu X.X., Yang X.J., Chao Y.L., Zheng H.M., Sheng H.F., Liu H.Y., He Y., Zhou H.W. (2017). The Potential Effect of Oral Microbiota in the Prediction of Mucositis During Radiotherapy for Nasopharyngeal Carcinoma. EBioMedicine.

[245] Sonis S.T. (2017). The Chicken or the Egg? Changes in Oral Microbiota as Cause or Consequence of Mucositis During Radiation Therapy. EBioMedicine.

[246] Cui M., Xiao H., Li Y., Zhou L., Zhao S., Luo D., Zheng Q., Dong J., Zhao Y., Zhang X. (2017). Faecal microbiota transplantation protects against radiation-induced toxicity. EMBO Mol. Med..

[247] Banerjee J., Mishra N., Dhas Y. (2015). Metagenomics: A new horizon in cancer research. Meta Gene.

[248] Martinez-Outschoorn U.E., Prisco M., Ertel A., Tsirigos A., Lin Z., Pavlides S., Wang C., Flomenberg N., Knudsen E.S., Howell A. (2011). Ketones and lactate increase cancer cell “stemness,” driving recurrence, metastasis and poor clinical outcome in breast cancer: Achieving personalized medicine via Metabolo-Genomics. Cell Cycle.

[249] Peiris-Pages M., Martinez-Outschoorn U.E., Pestell R.G., Sotgia F., Lisanti M.P. (2016). Cancer stem cell metabolism. Breast Cancer Res..

[250] Guppy M., Leedman P., Zu X., Russell V. (2002). Contribution by different fuels and metabolic pathways to the total ATP turnover of proliferating MCF-7 breast cancer cells. Biochem. J..

[251] Viale A., Corti D., Draetta G.F. (2015). Tumors and mitochondrial respiration: A neglected connection. Cancer Res..

[252] Alam M.M., Lal S., FitzGerald K.E., Zhang L. (2016). A holistic view of cancer bioenergetics: Mitochondrial function and respiration play fundamental roles in the development and progression of diverse tumors. Clin. Transl. Med..

[253] Shapira N. (2017). The potential contribution of dietary factors to breast cancer prevention. Eur. J. Cancer Prev..

[254] Keating E., Martel F. (2018). Antimetabolic Effects of Polyphenols in Breast Cancer Cells: Focus on Glucose Uptake and Metabolism. Front. Nutr..

[255] Backhed F., Ding H., Wang T., Hooper L.V., Koh G.Y., Nagy A., Semenkovich C.F., Gordon J.I. (2004). The gut microbiota as an environmental factor that regulates fat storage. Proc. Natl. Acad. Sci. USA.

[256] Sundaram S., Yan L. (2016). High-fat Diet Enhances Mammary Tumorigenesis and Pulmonary Metastasis and Alters Inflammatory and Angiogenic Profiles in MMTV-PyMT Mice. Anticancer Res..

[257] Maffei V.J., Kim S., Blanchard E.t., Luo M., Jazwinski S.M., Taylor C.M., Welsh D.A. (2017). Biological Aging and the Human Gut Microbiota. J. Gerontol. A Biol. Sci. Med. Sci..

[258] Wang W., Wang J., Li J., Yan P., Jin Y., Zhang R., Yue W., Guo Q., Geng J. (2018). Cholecystectomy Damages Aging-Associated Intestinal Microbiota Construction. Front. Microbiol..

[259] Kim S., Jazwinski S.M. (2018). The Gut Microbiota and Healthy Aging: A Mini-Review. Gerontology.

[260] Mahmoudian Dehkordi S., Arnold M., Nho K., Ahmad S., Jia W., Xie G., Louie G., Kueider-Paisley A., Moseley M.A., Thompson J.W. (2019). Altered bile acid profile associates with cognitive impairment in Alzheimer’s disease-An emerging role for gut microbiome. Alzheimers Dement..

[261] Grasset E., Puel A., Charpentier J., Collet X., Christensen J.E., Terce F., Burcelin R. (2017). A Specific Gut Microbiota Dysbiosis of Type 2 Diabetic Mice Induces GLP-1 Resistance through an Enteric NO-Dependent and Gut-Brain Axis Mechanism. Cell Metab..

[262] Liu R., Zhang C., Shi Y., Zhang F., Li L., Wang X., Ling Y., Fu H., Dong W., Shen J. (2017). Dysbiosis of Gut Microbiota Associated with Clinical Parameters in Polycystic Ovary Syndrome. Front. Microbiol..

[263] Chu H., Duan Y., Yang L., Schnabl B. (2019). Small metabolites, possible big changes: A microbiota-centered view of non-alcoholic fatty liver disease. Gut.

[264] Maruvada P., Leone V., Kaplan L.M., Chang E.B. (2017). The Human Microbiome and Obesity: Moving beyond Associations. Cell Host Microbe..

[265] Loomba R., Seguritan V., Li W., Long T., Klitgord N., Bhatt A., Dulai P.S., Caussy C., Bettencourt R., Highlander S.K. (2017). Gut Microbiome-Based Metagenomic Signature for Non-invasive Detection of Advanced Fibrosis in Human Nonalcoholic Fatty Liver Disease. Cell Metab..

[266] Mardinoglu A., Wu H., Bjornson E., Zhang C., Hakkarainen A., Räsänen S.M., Lee S., Mancina R.M., Bergentall M., Pietiläinen K.H. (2018). An Integrated Understanding of the Rapid Metabolic Benefits of a Carbohydrate-Restricted Diet on Hepatic Steatosis in Humans. Cell Metab..

[267] Lofterod T., Mortensen E.S., Nalwoga H., Wilsgaard T., Frydenberg H., Risberg T., Eggen A.E., McTiernan A., Aziz S., Wist E.A. (2018). Impact of pre-diagnostic triglycerides and HDL-cholesterol on breast cancer recurrence and survival by breast cancer subtypes. BMC Cancer.

[268] Miler J.J., Novotny P., Walker P.D., Harris J.R., MacLennan I.P. (1977). Neisseria gonorrhoeae and ABO isohemagglutinins. Infect. Immun..

[269] Potter E.V. (1982). Blood group antibodies induced by pneumococcal vaccine. J. Pediatr..

[270] Yadav S.K., Boppana S., Ito N., Mindur J.E., Mathay M.T., Patel A., Dhib-Jalbut S., Ito K. (2017). Gut dysbiosis breaks immunological tolerance toward the central nervous system during young adulthood. Proc. Natl. Acad. Sci. USA.

[271] Thevaranjan N., Puchta A., Schulz C., Naidoo A., Szamosi J.C., Verschoor C.P., Loukov D., Schenck L.P., Jury J., Foley K.P. (2017). Age-Associated Microbial Dysbiosis Promotes Intestinal Permeability, Systemic Inflammation, and Macrophage Dysfunction. Cell Host Microbe..

[272] Viaud S., Daillere R., Boneca I.G., Lepage P., Pittet M.J., Ghiringhelli F., Trinchieri G., Goldszmid R., Zitvogel L. (2014). Harnessing the intestinal microbiome for optimal therapeutic immunomodulation. Cancer Res..

[273] Zitvogel L., Ayyoub M., Routy B., Kroemer G. (2016). Microbiome and Anticancer Immunosurveillance. Cell.

[274] Li W., Han L., Yu P., Ma C., Wu X., Moore J.E., Xu J. (2014). Molecular characterization of skin microbiota between cancer cachexia patients and healthy volunteers. Microb. Ecol..

[275] Bindels L.B., Neyrinck A.M., Salazar N., Taminiau B., Druart C., Muccioli G.G., François E., Blecker C., Richel A., Daube G. (2015). Non Digestible Oligosaccharides Modulate the Gut Microbiota to Control the Development of Leukemia and Associated Cachexia in Mice. PLoS ONE.

[276] Bindels L.B., Neyrinck A.M., Claus S.P., Le Roy C.I., Grangette C., Pot B., Martinez I., Walter J., Cani P.D., Delzenne N.M. (2016). Synbiotic approach restores intestinal homeostasis and prolongs survival in leukaemic mice with cachexia. ISME J..

[277] Bischoff S.C. (2016). Microbiota and aging. Curr. Opin. Clin. Nutr. Metab. Care.

